# Unveiling the Biotechnological Potential of Cyanobacteria from the Portuguese LEGE-CC Collection Through Lipidomics and Antioxidant and Lipid-Lowering Properties

**DOI:** 10.3390/molecules30122504

**Published:** 2025-06-07

**Authors:** Flavio Oliveira, Tiago Conde, Marisa Pinho, Tânia Melo, Guilherme Scotta Hentschke, Ralph Urbatzka, Hugo Pereira, Monya Costa, Vitor Vasconcelos, Maria Rosário Domingues

**Affiliations:** 1 Interdisciplinary Centre of Marine and Environmental Research, CIIMAR/CIMAR LA, University of Porto, Terminal de Cruzeiros do Porto de Leixões, Av. General Norton de Matos s/n, 4450-208 Matosinhos, Portugal; oliveira_flavio@outlook.pt (F.O.); guilherme.scotta@gmail.com (G.S.H.); rurbatzka@ciimar.up.pt (R.U.); vmvascon@fc.up.pt (V.V.); 2 Biology Department, Faculty of Sciences, University of Porto, Rua do Campo Alegre 1021, 4169-007 Porto, Portugal; 3 Centre for Environmental and Marine Studies, CESAM, Department of Chemistry, Santiago University Campus, University of Aveiro, 3810-193 Aveiro, Portugal; marisapinho@ua.pt (M.P.); taniamelo@ua.pt (T.M.); mrd@ua.pt (M.R.D.); 4 GreenCoLab, Associação Oceano Verde, Universidade do Algarve, Campus de Gambelas, 8005-139 Faro, Portugal; hugopereira@greencolab.com (H.P.); monyacosta@greencolab.com (M.C.)

**Keywords:** antioxidant capacity, biotechnology, cyanobacteria, lipidomics, lipid-lowering capacity

## Abstract

Cyanobacteria are gram-negative prokaryotic microorganisms composed of both broad morphological and phylogenetic diversity inherited from diverse ecosystems like aquatic, terrestrial, or extremophilic environments. In this study, three cyanobacteria strains from the Blue Biotechnology and Ecotoxicology Culture Collection (LEGE-CC) were obtained from different environments in Portugal. Polyphasic analysis was applied for taxonomic identification. The proximate composition analysis indicated the lipid content (6.2% to 9.1% dry weight (DW)), protein content (28.2% to 62.9% DW), and carbohydrate content (19.5% to 46.1% DW). The fatty acid (FA) profiles of the strains revealed the presence of 19 different FAs, with FA 16:0 found in the highest abundance. The lipidomic analysis revealed 230 lipid species, with *Laspinema* sp. LEGE 06078 displaying the highest diversity (125 lipid species). These included species-specific and common lipids species that denote biochemical uniqueness that are also carriers of omega-3 FA (*n*−3). Biological assays exhibited strong antioxidant activity against ABTS^•+^ and DPPH^•^ in *Laspinema* sp. LEGE 06078, while *Sphaerospermopsis* sp. LEGE 00249 was renowned for reducing lipids in zebrafish larvae. The findings are of immense significance on the lipidomics diversity of cyanobacteria in terms of nutrition, health, and biotechnology, such as addressing obesity and sustainable resource production.

## 1. Introduction

Cyanobacteria, Gram-negative prokaryotic organisms, represent a diverse group of photosynthetic microorganisms with remarkable morphological and phylogenetic variability. They thrive across a wide range of ecological niches, including aquatic, terrestrial, and extremophilic environments, encompassing over 4500 species classified into 21 orders [1,2,3]. Portugal exhibits a diverse range of environments, such as volcanic islands, freshwater, estuarine and marine habitats, terrestrial areas, and salt flats. Recently, novel genera such as *Zarconia* [4], Venetifunis [5], *Pseudocalidococcus* [6], *Ciimarium* [7], and *Acaryochloriopsis*, *Pseudolimnococcus*, *Eucapsopsis*, and *Vasconcelosia* [8] have been described in these environments. Their type strains are maintained in culture collections such as The Blue Biotechnology and Ecotoxicology Culture Collection (LEGE-CC; https://lege.ciimar.up.pt/ (accessed on 28 January 2025)).

A few species of cyanobacteria have historically been consumed by humans due to their high nutritional value. Currently, EU-authorized products, according to the Novel Food Catalogue (NFC), include *Aphanizomenon flos*-*aquae* and *Limnospira platensis* (commercially known as spirulina), based on consumption history before 15 May 1997 [9,10]. Additionally, *Nostoc* sp. is commercially available in food markets in southern Peru and northern Chile communities [11] and has been part of the traditional Chinese diet since prehistoric times [12]. *L*. *platensis* and *Spirulina* sp. are widely distributed in Asia and have gained popularity in Western countries, with a market value estimated at USD 588.93 million in 2023, projected to reach USD 1192.20 million by 2030 [13]. This market expansion is driven by their bioactive properties, which make them attractive for various biotechnological applications [14]. These cyanobacteria are recognized for their high protein, antioxidant, and fatty acid (FA) content [12,14], potential beneficial effects against obesity [14,15], and incorporation into dietary supplements and food products [14]. Cyanobacteria can also be used as a source of bioactive compounds, such as in the case of dolastatin, which has been employed in the pharmaceutical industry as an antitumor agent [16].

Cyanobacteria are also major contributors to bioeconomy, providing sourcing feedstocks for different industrial sectors, such as food, feed, bioenergy, cosmetics, and bioplastics [17]. Several studies have explored the biotechnological potential of cyanobacteria, reporting on anti-cancer [18], anti-inflammatory [19], antibacterial and antifungal [20], and anti-adhesive coating properties [21]. For instance, Vega et al. [22] used different cyanobacteria extracts to evaluate the antioxidant and UV radiation-absorbing properties for cosmeceutical applications, while Tsai et al. [23] demonstrated that *Nostoc commune* has an anti-obesity effect and its use.

Obesity is one of the major burdens of our society, defined by the World Health Organization (WHO) as an abnormal or excessive accumulation of fat [24,25] that poses significant health risks. The WHO recognizes obesity as a global epidemic, contributing to more than 2.8 million deaths annually. In response, microalgae and cyanobacteria have emerged as potential alternatives for obesity management. These organisms are considered sustainable lipid sources (e.g., as substitutes to fish), particularly omega-3 (*n*−3) FA, which can enhance fat metabolism and reduce inflammation, thereby supporting weight management and metabolic health [15,26]. Ongoing research aims to identify safer and more effective therapeutic options, including natural sources like microalgae and cyanobacteria, which are primary producers and valuable sources of *n*−3 polyunsaturated FAs (PUFAs), with promising applications in disease prevention and treatment [27,28,29,30]. Furthermore, cyanobacteria are a source of essential FAs like linoleic acid omega-6 (*n*−6), which is responsible for promoting inflammatory and immune responses, as well as supporting the function of cell membranes [26]. Lipid analysis has been mostly dedicated to identifying FAs, e.g., *Gloeothece* sp. [31,32], *Phormidium* sp. [33], *Scytonema* sp. and *Oscillatoria* sp. [34]. However, cyanobacteria also contain other lipid classes.

The polar lipidome exhibits complex lipids such as phospholipids and glycolipids, where FAs are predominantly esterified. In recent years, increasing attention has been given to characterize the lipidome of microalgae and, to a lesser extent, cyanobacteria [35,36]. Some studies have elucidated the lipidome profile of cyanobacteria, including *Synechocystis* sp. [37,38], *Spirulina* sp. [39], and *Synechococcus* sp. PCC7002 [40], highlighting the uniqueness of lipid profiles among species. Additionally, environmental factors such as light intensity, temperature, and nutrient availability have been shown to significantly influence cyanobacterial lipid composition, demonstrating their structural and functional diversity [41,42]. To increase biomass production, for example, bioengineering has been used as a CRISPR strategy for promoting cyanobacteria faster growth [43]. Despite these insights, knowledge regarding the lipidomes of cyanobacteria remains limited, with most studies restricted to a few strains. It has also been shown that some of the complex lipids can contribute to boost the innovation and the market of cyanobacteria products by exploring the health benefits of such lipids [35,36]. An example of this is new products with lipid-lowering capacity, such as hydroxy-pheophytin identified in spirulina, which may support the development of novel nutraceuticals [44]. This knowledge gap has fueled interest in further lipidome investigations, aiming to identify high-value lipids for biotechnological applications.

In this context, the present study provides the first comprehensive analysis of the proximate composition and lipidome of three cyanobacterial strains from the LEGE-CC collection using liquid chromatography–high-resolution electrospray ionization mass spectrometry (LC-HR–ESI–MS). These strains, originating from diverse Portuguese environments, were assessed for their biotechnological potential through antioxidant activity evaluation and lipid-lowering assays in zebrafish larvae. A polyphasic approach was used to classify the strains: LEGE 06078 from a brackish water environment, LEGE 06114 from a marine habitat, and LEGE 00249 from freshwater. Additionally, the absence/presence of cyanotoxin-producing genes was determined to confirm their safety. These findings offer new insights into their polar lipid composition, highlighting their potential applications in nutrition (food and feed), cosmetics, and nutraceuticals.

## 2. Results

### 2.1. Phylogenetic Analysis and Potential for Cyanotoxin Production

**Figure 1:**
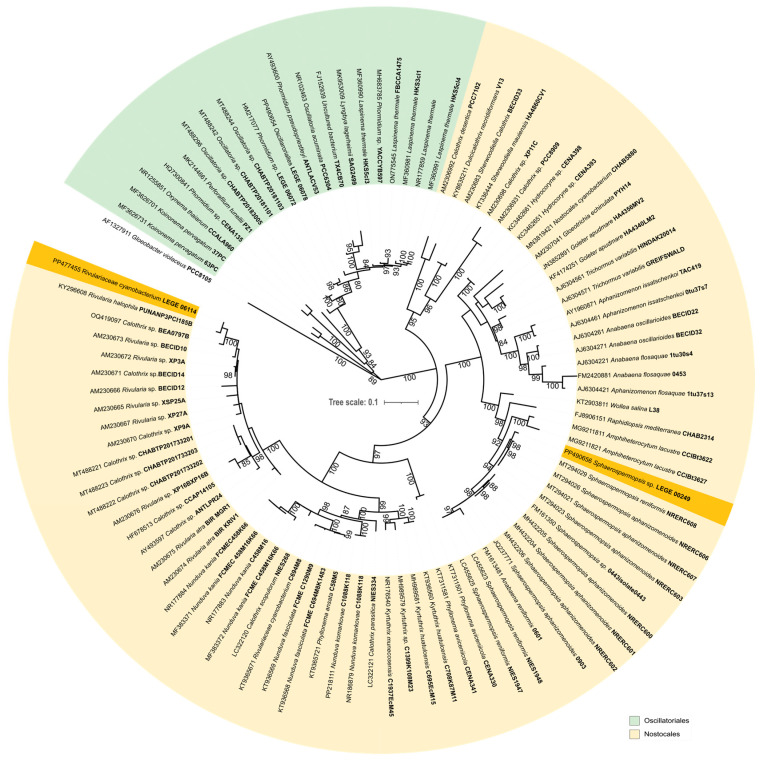
16S rRNA FastTree phylogenetic analysis of the studied strains and reference cyanobacteria strains. Numbers at the nodes represent bootstrap support values (%) based on 1000 replicates using the Maximum Likelihood (ML) method and bootstrap values lower than 80 were omitted from the figure. The strains highlighted represent described taxa. *Gloeobacter violaceus* PCC 8105 was used as the outgroup. The different color segments represent strain placement at the following order level.

Maximum-likelihood (ML) phylogenetic analysis (Figure 1) was performed, involving 95 nucleotide sequences and a total of 990 informative sites, which allowed us to separate the Oscillatoriales and Nostocales orders. Among the Oscillatoriales, strain LEGE 06078 was within the clade containing the *Laspinema* reference strain *Laspinema thermale* HK S5 (MF360990), with strong phylogenetic support (ML = 100), and both strains share 99% of 16S rRNA similarity. Based on this, LEGE 06078 is considered to belong to this genus. Previously, this strain was classified as *Phormidium* sp. [45]. Among the Nostocales, strain LEGE 06114 is phylogenetically related to the *Rivularia* reference strain *Rivularia halophila* PUNANP3PCI185B (KY296608), but with weak phylogenetic support (ML = 69). The similarity value between both strains is only 94.9%. These findings indicate that LEGE 06114 is phylogenetic distant to any other already described genus and cannot be classified within any currently described genus. For this reason, it should be described as a new genus in the future. Strain LEGE 00249 is also within the Nostocales, positioned within the clade containing the *Sphaerospermopsis* reference strain *Sphaerospermopsis aphanizomenoides* 04–43 (FM161350), with strong phylogenetic support (ML = 94). Both strains share 99.2% of 16S rRNA similarity. Based on this, LEGE 00249 is considered to belong to this genus (Figure 1). The study of the toxin-producing gene analysis revealed that none of the strains contained any of the analyzed toxin genes and are therefore considered safe for applications.

### 2.2. Morphological Characteristics of the Cyanobacteria Strains

The morphological analysis revealed that *Laspinema* sp. LEGE 06078 exhibits filaments that can be either straight or wavy, with thin sheaths. The trichomes are cylindrical, unbranched, isopolar, motile, and slightly constricted at cross walls. The cells are shorter than wide, with blue-green or olive-green content. The terminal cell is elongated, either conical, straight or bent, with a rounded end without a calyptra. These characters are consistent with the circumscription of *Laspinema*, in agreement with the phylogenetic and similarity results.

Strain *Rivulariaceae cyanobacterium* LEGE 06114 is characterized by long filaments that are solitary or form macroscopic clusters. The filaments are waved or coiled, with a firm and colorless sheath, which is sometimes slightly lamellated and open at the apex. The trichomes are false-branched, with facultative constrictions and attenuation toward the ends. The cells are cylindrical or barrel-like, shorter than wide, with granular, blue-green, olive-green, or gray-blue content. End cells are widely rounded, sometimes capitate, or have slightly thickened outer cell walls. Heterocysts are present. *Rivulariaceae cyanobacterium* LEGE 06114 differs from *Rivularia*, the phylogenetically closest related genera, by presenting slightly attenuated or not attenuated trichomes, while *Rivularia* markedly presents attenuation of the trichomes toward the ends. This strain is likely to represent a new cyanobacterial genus that should be described in the future.

*Sphaerospermopsis* sp. LEGE 00249 features coiled or straight trichomes, which can be constricted, with a facultatively present sheaths. The terminal cells are elongated and tapered or undifferentiated. Vegetative cells, spherical or barrel-shaped, contain aerotopes and are compressed during division. Heterocysts are intercalary, spherical, and solitary, while akinetes are spherical or nearly spherical, located adjacent to one or both sides of the heterocysts. These characters are consistent with the circumscription of *Sphaerospermopsis*, in agreement with the phylogenetic and similarity results.

### 2.3. Biomass Proximate Composition Analysis

**Figure 2:**
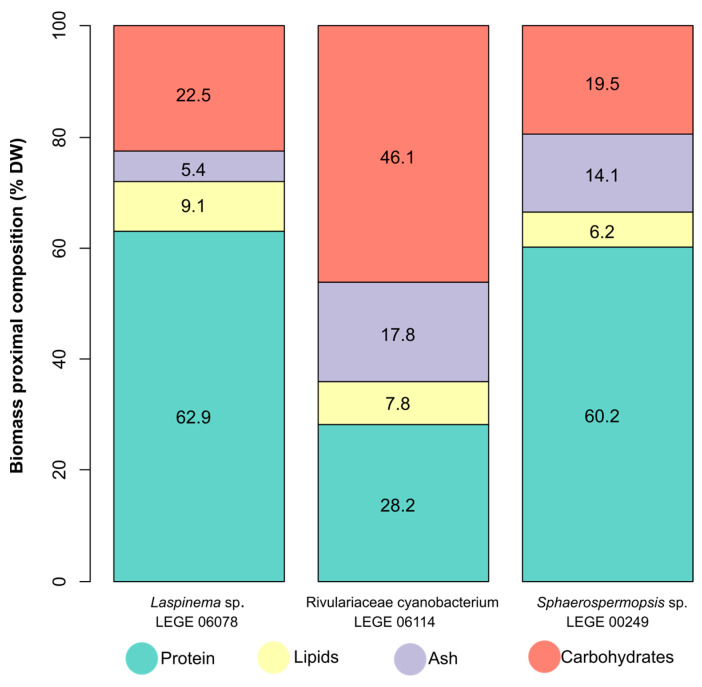
Biomass proximate composition of the cyanobacteria strains (*n* = 3) in % DW. Different colors indicate different compositions of biomass (protein in green, lipids in yellow, ash in purple, and carbohydrates in red).

The proximate composition analysis of biomass is shown in Figure 2 (Supplementary Figures S2 and S3 and Table S1). Ash content was lowest for *Laspinema* sp. LEGE 06078 (5.4% dry weight (DW)) and similar between *Rivulariaceae cyanobacterium* LEGE 06114 (17.8% DW) and *Sphaerospermopsis* sp. LEGE 00249 (14.1% DW). The lowest protein content was observed for *Rivulariaceae cyanobacterium* LEGE 06114 (28.2% DW), and the highest protein content was observed for *Laspinema* sp. LEGE 06078 (62.9% DW) and *Sphaerospermopsis* sp. LEGE 00249 (60.2% DW). The statistical analysis of protein content (Supplementary Figure S2) showed significant differences between the groups *Laspinema* sp. LEGE 06078 and *Rivulariaceae cyanobacterium* LEGE 06114 (*p* = 0.002), and between the group *Rivulariaceae cyanobacterium* LEGE 06114 and *Sphaerospermopsis* sp. LEGE 00249 (*p* = 0.021).

The lowest lipid content was observed for *Sphaerospermopsis* sp. LEGE 00249 (6.2% DW), and the highest lipid content was observed for *Laspinema* sp. LEGE 06078 (9.1% DW). *Rivulariaceae cyanobacterium* LEGE 06114 showed 7.8% DW of lipid content. The statistical analysis of total lipid content (Supplementary Figure S3) showed results with significant differences between the groups *Laspinema* sp. LEGE 06078 and *Sphaerospermopsis* sp. LEGE 00249 (*p* = 0.009). The lowest carbohydrate content was similar for *Laspinema* sp. LEGE 06078 (22.5% DW) and *Sphaerospermopsis* sp. LEGE 00249 (19.5% DW), and the highest for *Rivulariaceae cyanobacterium* LEGE 06114 (46.1% DW).

Non-metric multidimensional scaling (NMDS) detected dissimilarity between the cyanobacteria strains (Supplementary Figure S4), presenting a low stress value (provides an excellent representation in reduced dimensions). This analysis showed that the biomass composition of Rivulariaceae cyanobacterium LEGE 06114 is associated with high relative ash and carbohydrates, but its higher carbohydrate content showed a displacement toward this component in the first dimension. *Sphaerospermopsis* sp. LEGE 00249 was characterized by ash and protein. This strain showed a balanced proportion between these two components. *Laspinema* sp. LEGE 06078 is associated with high relative protein and lipids, but its higher lipids content showed a displacement toward this component in the first dimension. *Laspinema* sp. LEGE 06078 and *Sphaerospermopsis* sp. LEGE 00249 were associated with protein since they had the largest proportion.

### 2.4. Fatty Acid Composition and Total Lipid Content of Cyanobacteria Strains

This analysis allowed the identification of 19 FAs across the cyanobacteria (Table 1 and Table S2). FA 16:0 was the most abundant FA in all the strains, accounting for 37.7 ± 1.8% (*Laspinema* sp. LEGE 06078), 29.0 ± 2.3% (*Rivulariaceae cyanobacterium* LEGE 06114), and 25.1 ± 3.0% (*Sphaerospermopsis* sp. LEGE 00249). Other FAs were found in notable abundance in the following strains: *Laspinema* sp. LEGE 06078, which showed FA 18:4 *n*−3 (10.4 ± 0.3%), followed by FA 18:1 *n*−9 (9.0 ± 0.6%) and FA 18:3 *n*−3 (8.9 ± 0.3%). In Rivulariaceae cyanobacterium LEGE 06114, FA 18:3 *n*−6 (12.3 ± 1.2%), FA 18:1 *n*−9 (10.3 ± 1.3%), and FA 18:2 *n*−6 (9.5 ± 0.2%) were observed with high abundance. Lastly, *Sphaerospermopsis* sp. LEGE 00249 exhibited FA 18:0 (15.1 ± 1.4%), FA 18:3 *n*−3 (11.0 ± 1.2%), and FA 18:2 *n*−6 (10.2 ± 1.1%). Some FAs were only observed in one or two strains, e.g., FA 17:0 and FA 17:1 *n*−8 present only in *Laspinema* sp. LEGE 06078, or FA 16:1 *n*−5, FA 16:2 *n*−4, and FA 16:3 *n*−3 present only in *Rivulariaceae cyanobacterium* LEGE 06114. Another example is FA 16:1 *n*−9 present in *Laspinema* sp. LEGE 06078 and Rivulariaceae cyanobacterium LEGE 06114, but not in *Sphaerospermopsis* sp. LEGE 00249. Principal component analysis (PCA) was conducted to explore the relationships between the FA profiles of strains (Supplementary Figure S5), and the results of PC1 explain 55.6% of the total variance. The complete linkage cluster analysis of the FA profile identified two chemotype clusters (Supplementary Figure S6). Strain *Sphaerospermopsis* sp. LEGE 00249 was clearly separated from two other cyanobacteria in the complete linkage cluster analysis, which displayed a substantial difference between clusters. It is in good agreement with the results of the FA profile and PCA. On the other hand, the chemotype cluster shows that the cyanobacteria have been grouped by FA, and interestingly, *Laspinema* sp. LEGE 06078 and *Rivulariaceae cyanobacterium* LEGE 06114 belong to the same cluster but of a different order (*Oscillatoriales* and *Nostocales*, respectively).

The potential health benefits of the strains atherogenic index (AI), thrombogenic index (TI), hypocholesterolemic/hypercholesterolemic ratio index, and *n*−6/*n*−3 ratio are described in Table 1. The AI of the lipid extracts was 0.9%, 0.6%, and 1.0% for *Laspinema* sp. LEGE 06078, *Rivulariaceae cyanobacterium* LEGE 06114, and *Sphaerospermopsis* sp. LEGE 00249, respectively. The TI was 0.6%, 0.7%, and 0.9% for *Laspinema* sp. LEGE 06078, *Rivulariaceae cyanobacterium* LEGE 06114, and *Sphaerospermopsis* sp. LEGE 00249, respectively. Meanwhile, the h/H ratio was 0.6%, 0.8%, and 0.9% for *Laspinema* sp. LEGE 06078, *Rivulariaceae cyanobacterium* LEGE 06114, and *Sphaerospermopsis* sp. LEGE 00249, respectively, and the *n*−6/*n*−3 ratio was 0.4%, 2.4%, and 0.9% for *Laspinema* sp. LEGE 06078, *Rivulariaceae cyanobacterium* LEGE 06114, and *Sphaerospermopsis* sp. LEGE 00249, respectively.

### 2.5. Identification of the Lipidome of the Cyanobacteria Strains

The lipid profile of cyanobacterial strains was identified, and four main groups of lipids were identified, namely glycolipids (GLs), phospholipids (PLs), betaine lipids (BLs), and neutral lipids (NLs), which included different lipid classes, as shown in Table 2. These included 125 in *Laspinema* sp. LEGE 06078, 106 in *Rivulariaceae cyanobacterium* LEGE 06114, and 113 in *Sphaerospermopsis* sp. LEGE 00249. Overall, a total of 230 different lipid species were assigned. The total number of lipid species and the number of lipid species per class are also shown in Table 2. *Laspinema* sp. LEGE 06078 was the cyanobacteria with the highest number of different lipid species, and the species with the lowest was *Sphaerospermopsis* sp. LEGE 00249.

**Figure 3:**
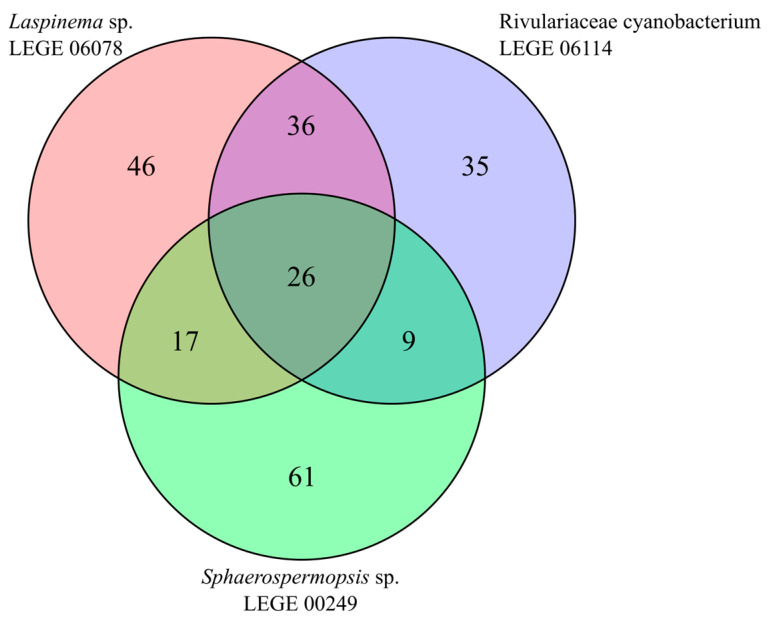
Venn diagram of the total number of lipid species. Numbers within the diagram represent the number of lipid species shared between the corresponding groups.

The comparison of lipid profiles among the cyanobacteria in this study revealed that from the total of 230 lipid species identified, only 26 lipid species (Table 3) were common to all three lineages, as can be seen in the Venn diagram plotted (Figure 3). Of these 26 lipid species, 19 were GLs (7 DGDGs, 8 MGDGs, 4 SQDGs), 3 were PLs (1 PC, 2 PGs), and 4 were neutral lipids (TGs) (Table 3). This suggests that cyanobacteria have a species-specific polar lipid signature. Also, there are several lipid species only detected in a specific strain, namely 46 for *Laspinema* sp. LEGE 06078, 35 for *Rivulariaceae cyanobacterium* LEGE 06114, and 61 for *Sphaerospermopsis* sp. LEGE 00249 (Figure 3).

**Figure 4:**
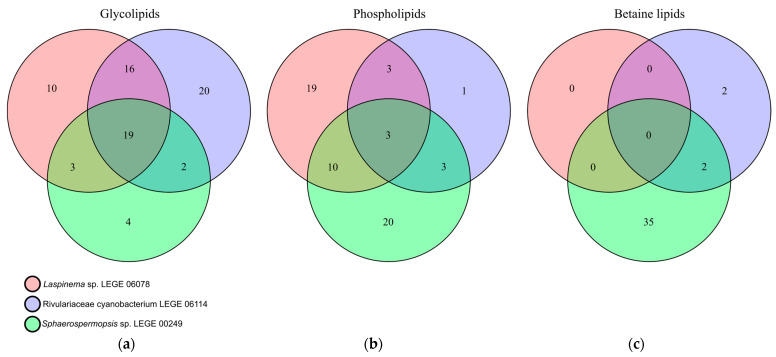
Venn diagram. Distribution of lipids between cyanobacteria strains. (**a**) Glycolipids; (**b**) phospholipids; (**c**) betaine lipids.

The GL composition included the classes monogalactosyldiacylglycerol (MGDG), digalactosyldiacylglycerols (DGDGs), and sulfoquinovosyl diacylglycerols (SQDGs), detected in all strains, but with dissimilar composition. The diacylglyceryl-α-D-glucuronide (DGGA) was detected only in *Rivulariaceae cyanobacterium* LEGE 06114. These classes have different numbers of lipid species depending on the strain, as can be seen in Table 2. *Laspinema* sp. LEGE 06078 had 48 lipid species, while *Rivulariaceae cyanobacterium* LEGE 06114 had 57, and *Sphaerospermopsis* sp. LEGE 00249 had 28. Also, as plotted on the Venn diagram of the GL species, there are some that are common, while others are specific to a strain (Figure 4a). This gives evidence that cyanobacteria have a species-specific GL profile, but also that there are several lipid species only detected in a specific strain, namely 10 for *Laspinema* sp. LEGE 06078, 20 for *Rivulariaceae cyanobacterium* LEGE 06114, and 4 for *Sphaerospermopsis* sp. LEGE 00249. The profile within each class is also variable, and the most abundant MGDG was MGDG (34:4) for *Laspinema* sp. LEGE 06078 and *Sphaerospermopsis* sp. LEGE 00249, and MDGD (34:3) for *Rivulariaceae cyanobacterium* LEGE 06114. The most abundant DGDG was DGDG (34:4) for *Laspinema* sp. LEGE 06078, and DGDG (34:3) for strains *Rivulariaceae cyanobacterium* LEGE 06114 and *Sphaerospermopsis* sp. LEGE 00249 (Table 2 and Supplementary Figure S7). DGGA was exclusively detected in *Rivulariaceae cyanobacterium* LEGE 06114, with DGGA (35:1) being the most abundant species. The most abundant SQDG was SQDG (34:1) for *Laspinema* sp. LEGE 06078 and *Rivulariaceae cyanobacterium* LEGE 06114, and SQDG (34:3) for *Sphaerospermopsis* sp. LEGE 00249 (Table 2 and Table S3).

The PL composition included the classes phosphatidylcholine (PC), phosphatidylethanolamine (PE), phosphatidylinositol (PI), phosphatidylglycerol (PG), and lyso forms (LPC, LPG, and LPE). These classes have different numbers of lipid species depending on the strain, as can be seen in Table 2. *Laspinema* sp. LEGE 06078 contains 35 lipid species, while *Rivulariaceae cyanobacterium* LEGE 06114 has 10, and *Sphaerospermopsis* sp. LEGE 00249 has 36. As shown in the Venn diagram of the PL species, there are some that are common, while others are specific to each strain (Figure 4b). This gives evidence that cyanobacteria have a species-specific PL, but also that there are several lipid species only detected in a specific strain, namely 19 for *Laspinema* sp. LEGE 06078, 1 for *Rivulariaceae cyanobacterium* LEGE 06114, and 20 for *Sphaerospermopsis* sp. LEGE 00249. The profile within each class is also variable (Supplementary Figure S6), and the most abundant PC was PC (36:2) in all cyanobacteria. The most abundant PE was PE (36:2) for *Laspinema* sp. LEGE 06078, PE (30:0) for *Rivulariaceae cyanobacterium* LEGE 06114, and PE (32:1) for *Sphaerospermopsis* sp. LEGE 00249. The most abundant PG and common for all cyanobacteria was PG (34:1). In the case of the PI class, PI (34:1) was the most abundant for *Laspinema* sp. LEGE 06078 and *Sphaerospermopsis* sp. LEGE 00249. Regarding the lyso forms, the most abundant LPC was LPC (18:1) for *Laspinema* sp. LEGE 06078 and *Sphaerospermopsis* sp. LEGE 00249. Only one LPE, LPE (18:1), was observed in *Sphaerospermopsis* sp. LEGE 00249 and. The LPG was only observed in *Sphaerospermopsis* sp. LEGE 00249 with one lipid species LPG (16:0) (Supplementary Figure S6).

The BL composition included diacylglyceryl-N,N,N-trimethyl-homoserine (DGTS) and monoacylglyceryl-N,N,N-trimethyl-homoserine (MGTS). These classes have a different number of lipid species depending on the strain, as can be seen in Table 2. BLs were present in *Rivulariaceae cyanobacterium* LEGE 06114 with 4 lipid species, and *Sphaerospermopsis* sp. LEGE 00249 with 37 lipid species. DGTS was not detected in *Laspinema* sp. LEGE 06078. As shown in the Venn diagram of the BL species, there are none that are common, while others are specific to each strain (Figure 4c). This provides evidence that cyanobacteria have a species-specific BL, but also that there are several lipid species only detected in a specific strain, namely 2 for *Rivulariaceae cyanobacterium* LEGE 06114, and 35 for *Sphaerospermopsis* sp. LEGE 00249. The most abundant DGTS was DGTS (36:2) for *Rivulariaceae cyanobacterium* LEGE 06114, and DGTS (34:3) for *Sphaerospermopsis* sp. LEGE 00249 (Supplementary Figure S7). The lyso form of the betaines, the MGTS, was identified only in *Sphaerospermopsis* sp. LEGE 00249 with the most abundant species MGTS (16:0) (Supplementary Figure S7).

The neutral lipids identified were the triacylglycerols (TGs), present in all cyanobacteria, and the diacylglycerols (DG); however, they were only found in *Laspinema* sp. LEGE 06078 and *Rivulariaceae cyanobacterium* LEGE 06114 (Table 2 and Table S3). The most abundant TGs were TG (50:1), TG (48:0), and TG (52:2) for *Laspinema* sp. LEGE 06078, *Rivulariaceae cyanobacterium* LEGE 06114, and *Sphaerospermopsis* sp. LEGE 00249, respectively (Supplementary Figure S7). DG showed the most abundant DG (34:4) and DG (34:3) for *Laspinema* sp. LEGE 06078 and *Rivulariaceae cyanobacterium* LEGE 06114, respectively (Supplementary Figure S7).

**Figure 5:**
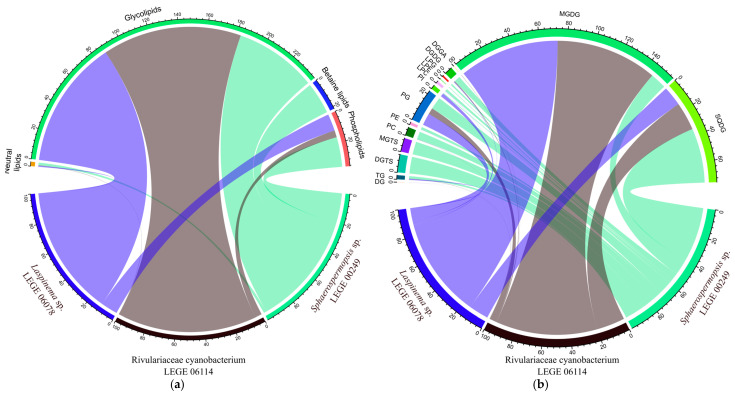
Graphical representation of the relative abundance of polar lipids distributed over the studied cyanobacteria. Different colors represent different cyanobacterial strains. (**a**) Glycolipids (GLs), phospholipids (PLs), betaines lipids (BLs), and neutral lipids (NLs); (**b**) classes of lipids.

The graphical representation of the distribution of polar lipids among the cyanobacteria studied (Figure 5a) shows that GLs were similarly distributed between *Laspinema* sp. LEGE 06078 and *Rivulariaceae cyanobacterium* LEGE 06114, but PLs were mainly present in *Sphaerospermopsis* sp. LEGE 00249 and lowest in *Rivulariaceae cyanobacterium* LEGE 06114. BLs were more expressed in *Sphaerospermopsis* sp. LEGE 00249, but not present in *Laspinema* sp. LEGE 06078, and less present in *Rivulariaceae cyanobacterium* LEGE 06114. Considering the distribution of the classes of GLs, PLs, and BLs (Figure 5b), the MGDG was mostly exhibited in *Laspinema* sp. LEGE 06078 and *Rivulariaceae cyanobacterium* LEGE 06114, while SQDG was more exhibited in *Rivulariaceae cyanobacterium* LEGE 06114. For *Sphaerospermopsis* sp. LEGE 00249, the most exhibited was SQDG (GL), and the second most exhibited, was DGTS (BL).

### 2.6. In Chemico Evaluation of Antioxidant Activity

**Figure 6:**
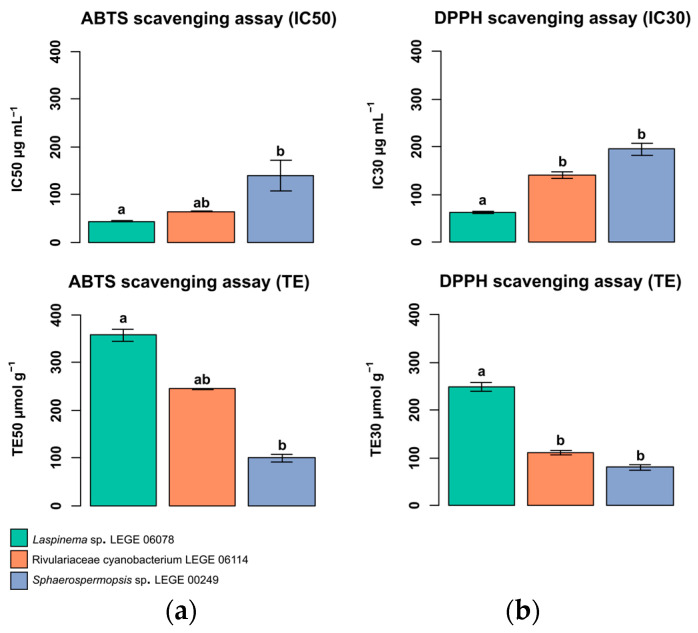
Evaluation of the antioxidant activity of cyanobacterial lipid extracts from this study. Concentration of lipid extract (µg mL^−1^) that provided the following: (**a**) 50% inhibition of the ABTS^•+^ radical and (**b**) 30% inhibition of the DPPH^•^ radical. Trolox equivalents are expressed as TE µmol g^−1^. The values are displayed as the mean (*n* = 3) ± standard deviation. Different letters represent significant differences between cyanobacteria strains.

The antioxidant activity of the lipid extracts from cyanobacterial strains are presented in Figure 6. The lipid extracts from the cyanobacteria exhibited 50% inhibition (IC50) in the 2,2′-azino-bis-(3-ethylbenzothiazoline-6-sulfonic acid) radical cation (ABTS^•+^) assay at the tested concentrations. The lowest IC50 was obtained for *Laspinema* sp. LEGE 06078 at 43.6 ± 1.1 μg mL^−1^ and Trolox equivalents (TEs) of 357.2 ± 9.1 µmol g^−1^. *Rivulariaceae cyanobacterium* LEGE 06114 showed an IC50 value of 63.8 ± 0.1 μg mL^−1^ and TE of 244.3 ± 0.2 µmol g^−1^. *Sphaerospermopsis* sp. LEGE 00249 had the highest value of 157.1 ± 8.0 μg mL^−1^ and a TE of 99.4 ± 5.1 µmol g^−1^. Statistically, the only evident differences were observed when comparing *Laspinema* sp. LEGE 06078 and *Sphaerospermopsis* sp. LEGE 00249 with *p* = 0.01 (Figure 6a).

The 2,2-diphenyl-1-picrylhydrazyl radical (DPPH^•^) assay showed 30% inhibition (IC30) of radicals for all lipid extracts. The lowest IC30 was observed for *Laspinema* sp. LEGE 06078 at 62.8 ± 2.0 μg mL^−1^ and TE of 249.3 ± 7.7 µmol g^−1^. *Rivulariaceae cyanobacterium* LEGE 06114 presents values of 141.0 ± 5.8 μg mL^−1^ and TE of 111.0 ± 4.5 µmol g^−1^. The highest value was for *Sphaerospermopsis* sp. LEGE 00249, with an IC30 of 195.2 ± 8.4 μg mL^−1^ and TE of 80.3 ± 3.5 µmol g^−1^. Statistically, *Laspinema* sp. LEGE 06078 differed significantly from *Rivulariaceae cyanobacterium* LEGE 06114 and *Sphaerospermopsis* sp. LEGE 00249 (*p* = 0.03 and *p* < 0.01, respectively) (Figure 6b).

### 2.7. Lipid-Lowering Activity

**Figure 7:**
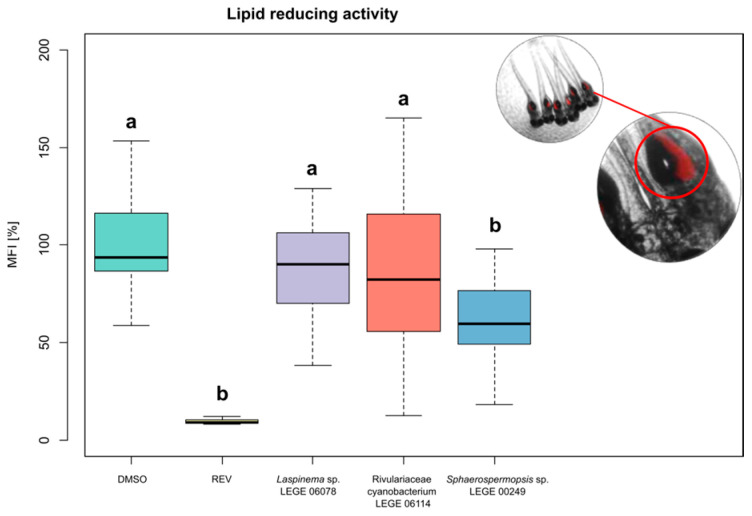
Zebrafish Nile red fat metabolism assay. Box–whisker plots of lipid-lowering activity in zebrafish larvae after 48 h exposure to lipid extracts (10 μg mL^−1^) with representative images of the assay (fluorescence channel and overlay of dark field). Solvent control was 1% dimethyl sulfoxide (DMSO), and positive control was 50 μM resveratrol (REV). Values are expressed as the percentage of mean fluorescence intensity (MFI) relative to the DMSO group and are derived from five to six individual larvae per cyanobacterial lipid extract (*n* = 30) from two independent experiments. Letters highlight significant altered fluorescence intensities vs. the solvent control (DMSO), indicating changes in neutral lipid level (*p* ≤ 0.001).

The fluorescence microscopy and quantitative analysis of Nile red provided evidence for the lipid-lowering capacity of the *Sphaerospermopsis* sp. LEGE 00249 lipid extract (*p* < 0.05) in zebrafish larvae, with a significant decrease in lipids in the yolk sac at 41% of Nile red staining at 10 μg mL^−1^ when compared to the solvent control (*p* < 0.001), as shown in Figure 7. When comparing the lipid extract of *Laspinema* sp. LEGE 06078 and *Rivulariaceae cyanobacterium* LEGE 06114 with DMSO, no statistically significant differences were observed (*p* > 0.05). This reduction suggests a potential lipid-lowering capacity of this cyanobacterial lipid extract, using zebrafish larvae as a model. Resveratrol (REV) was used as a positive control at a final concentration of 50 μM, also with significantly reduced Nile red lipid staining (*p* < 0.001), validating the sensitivity of the assay. The solvent control was 1% dimethyl sulfoxide (DMSO), which did not cause any observable toxicity or malformations in zebrafish larvae. To assess the safety profile of the lipid extracts, toxicity assays were performed by evaluating the larval mortality at 24 h and 48 h post exposure, and monitoring morphological abnormalities, such as pericardial edema, yolk sac edema, or spinal deformities. In our study, no adverse effects or malformations were observed for any of the tested lipid extracts.

## 3. Discussion

Cyanobacteria are organisms with a wide taxonomic variety, and they may inhabit various environments, ranging from marine to terrestrial habitats. Their complex phylogeny has been the focus of intense research due to their significant biotechnological potential. Cyanobacteria are capable of producing nutrient-rich compounds, aligning with global initiatives like those of the United Nations to promote sustainable food sources. They are particularly abundant in proteins, carbohydrates, and essential lipids such as *n*−3 and *n*−6 FAs, making them a valuable addition to a healthy and sustainable diet.

Currently, both human and animal diets often lack essential FAs, leading to health issues such as cardiovascular diseases and diabetes. In this context, cyanobacteria hold promises for improving the nutritional quality of foods while providing nutritional and bioactive compounds, as is well known for the spirulina, widely consumed worldwide. In this study, three cyanobacteria were selected and cultivated under autotrophic conditions to explore their biochemical and nutritional properties. Additionally, their bioactive properties and potential health effects were assessed through various in vivo and in vitro assays.

Firstly, the taxonomic position of the species was assessed by phylogenetic analysis (Figure 1) using the 16S rDNA region from the three cyanobacteria. The results distinguished two orders: Nostocales (*Rivulariaceae cyanobacterium* LEGE 06114 and *Sphaerospermopsis* sp. LEGE 00249) and Oscillatoriales (*Laspinema* sp. LEGE 06078) [1]. Morphologically, these cyanobacteria are distinct from each other. Our ML 16S rRNA phylogenies, which included reference strains from both orders as well genera from the Oscillatoriales and Nostocales, corroborated the findings of May et al. [46] and Strunecký et al. [1]. This study contributes to the understanding of biotechnological potential within taxonomic diversity. Although these cyanobacteria are not approved for human consumption according to Novel Food by the European Food Safety Authority (EFSA), cyanobacteria-based products have gained importance due to recent investigations into their use as dietary supplements for food or feed, as well as for disease treatment. Accurate taxonomic classification allows for better understanding and comparison of different studies, contributing to advancements in the European cyanobacteria and microalgae industry and the scientific field.

The demand for alternative and sustainable sources of food, supplements, and bioactive compounds has placed cyanobacteria in the spotlight. To understand the nutritional potential of cyanobacteria, it is necessary to evaluate the biomass composition, providing an overview of its biochemical profile. The total protein content did not show significant differences between the freshwater cyanobacteria *Laspinema* sp. LEGE 06078 and *Sphaerospermopsis* sp. LEGE 00249. However, when compared to the marine cyanobacterium *Rivulariaceae cyanobacterium* LEGE 06114, differences were observed. When compared to other cyanobacterial genera studied, such as *Spirulina* [14], *Anabaena*, and *Aphanizomenon* [47], the values presented are similar. The marine cyanobacterium *Rivulariaceae cyanobacterium* LEGE 06114 showed a lower protein content (28.2% DW), which may be related to its higher production of carbohydrates (46.1%), associated with the physiological mechanism to maintain osmotic stability and cellular functionality [48]. Environmental factors, such as variation in salinity, temperature, and nutrient availability, play a crucial role in lipid profile, especially in mechanisms of membrane regulation and oxidative stress [49]. When the cultivation is performed in laboratory, under standard conditions, these environmental factors were not present during the cultivation process, but the genetic signatures of environmental adaptation are persistent, like a physiological mechanism to maintain osmotic stability [50,51]. Similar protein content was reported for marine *Cyanobium* sp. LEGE 06140 (24% DW) [52]. This is not observed in freshwater cyanobacteria, which have carbohydrate values of 22.5% and 19.5% for *Laspinema* sp. LEGE 06078 and *Sphaerospermopsis* sp. LEGE 00249, respectively. A study by Aouir et al. [53] demonstrated that *Arthrospira platensis* (in powder and lyophilized form) from different countries has a protein content ranging from 23.8% to 62.7%.

In our study, the total lipid content varied between 6.2% DW and 9.1% DW. Statistically significant differences were observed only between freshwater cyanobacteria. Studies on the commercially significant cyanobacteria *Spirulina* sp. and *S. platensis* have reported lipid values ranging from 1.5% to 8.1% [14,53], while another study by Conde et al. [26] with *Spirulina* sp. reported a total lipid content of 10.7% of biomass. A study with *Cyanobium* sp. LEGE 15611, conducted by Cruz et al. [52], revealed higher lipid content (12% DW) compared to our findings. Variations in lipid content among cyanobacteria may be associated with a complex combination of factors such as nutrient availability, light intensity, oxidative stress, and others. However, it should be noted that total lipid contents vary between species and depend on the extraction methodology used. Additionally, using raw organic extracts from published works may include other molecules beyond lipids, which can contribute to the weight of the extract higher than the one reported in our work, which correspond to a pure lipid extract [54,55].

The FA profiles were evaluated, and despite some common FAs, each cyanobacterium exhibited a unique FA signature (Table 1 and Table S2), with variations in relative abundance as observed in the PCA (Supplementary Figure S5) and hierarchical clustering analysis (Supplementary Figure S6). When analyzing the freshwater cyanobacterium *Laspinema* sp. LEGE 06078 individually, no prior studies on its FA profile have been described. However, when compared with a taxonomically close cyanobacterium within the same family, *Perforafilum tunnelli* (National Center for Biotechnology Information—NCBI accession number: MK214466), a study by Zimba et al. [56] identified 11 FAs. The FA profile of *P. tunnelli* and *Laspinema* sp. LEGE 06078 is similar when comparing SFAs, except for the presence of FA 14:0 in *Laspinema* sp. LEGE 06078. In terms of MUFAs, some FAs present in *P. tunnelli* are absent in *Laspinema* sp. LEGE 06078 (e.g., FA 17:1 and FA 18:1), and also observed in reverse (e.g., FA 17:1 *n*−8 and FA 18:1 *n*−7). The PUFAs between the two species are identical, except for the presence of FA 22:6 *n*−3 in *P. tunnelli*.

For the marine cyanobacterium *Rivulariaceae cyanobacterium* LEGE 06114 and for cyanobacterium within this genus and the Rivulariaceae family, no studies have referenced the FA profile. In the Nostocales order, research on genera such as *Aphanizomenon* (Aphanizomenonaceae family) and *Calothrix* (Calotrichaceae family) has highlighted differences in FA profiles [57,58]. These differences may be associated with their taxonomic family distinctions or factors such as cultivation conditions, extraction methods, and FA quantification/identification. Nonetheless, all cyanobacterial species studied exhibit FA compositions ranging from 14 to 18 carbon atoms.

Among the cyanobacteria studied in this work, *Sphaerospermopsis* sp. LEGE 00249 is the most documented in the literature [59,60,61,62]. Moretto et al. [63] evaluated different extraction methods for *Sphaerospermopsis torques* and identified only six FAs. When comparing our results with this study, the most significant difference observed is in the relative abundance (%) of MUFAs and PUFAs (in the extract obtained using traditional extraction method), where the composition of *Sphaerospermopsis* sp. LEGE 00249 is higher (22% and 21%, respectively). However, in the same study, microwave-assisted extraction yielded a higher PUFA value than found in our study (36%). The FA profile identified by Zapomělová et al. [64] is similar to those reported in our study. The hierarchical clustering analysis (Supplementary Figure S6) illustrates that the FA profile of this cyanobacterium is notably distinct from the other two cyanobacterial species in this study. One characteristic is the lower number of FAs identified compared to the other two species.

The FA cyanobacteria variation is a dynamic process that allows adaptation to a wide range of growth conditions. In our study, we observed FA compositions with up to 18 carbon atoms, except for cyanobacterium *Sphaerospermopsis* sp. LEGE 00249, which also exhibited an FA with 24 carbon atoms. Some studies have reported FAs with more than 18 carbon atoms, such as in the genera *Anabaena*, *Microcystis*, and *Spirulina* [65]. Another study by Dembitsky et al. [66] with the genus *Aphanizomenon* identified saturated FAs with 4 to 12 carbon atoms.

Considering the cyanobacterial strains analyzed in this study, the *n*−3 FAs present in *Rivulariaceae cyanobacterium* LEGE 06114 and *Sphaerospermopsis* sp. LEGE 00249 showed relative abundances of 11.36% and 10.96%, respectively, while *Laspinema* sp. LEGE 06078 had a higher relative abundance of 19.31%. Other genera also exhibited *n*−3 FAs, such as *Anabaena*, *Aphanizomenon*, *Microcystis*, *Nostoc*, and *Planktothrix* [57], in varying amounts (between 5.2% and 41.4%) depending on cultivation conditions. The presence of *n*−3 FAs has been observed in these genera and others, which is not the case for *Spirulina* sp. Some studies report the absence of *n*−3 FAs in *Spirulina* sp. [26,67,68], which may be associated with chemotaxonomic markers of this species. *n*−6 FAs has shown values of 8.8%, 26.8%, and 10.2% for *Laspinema* sp. LEGE 06078, *Rivulariaceae cyanobacterium* LEGE 06114, and *Sphaerospermopsis* sp. LEGE 00249, respectively. These values are lower compared to that for *Spirulina* sp. (44.6% *n*−6) in a study by Conde et al. [26]. In contrast, a study with *Mastigocladus laminosus* reported values ranging from 0.4% to 1.9% [69]. Notably, in terms of relative abundance, *Laspinema* sp. LEGE 06078 highlighted stearidonic acid (SDA), while *Sphaerospermopsis* sp. LEGE 00249 had alpha-linolenic acid (ALA), and *Rivulariaceae cyanobacterium* LEGE 06114 had gamma-linolenic acid (GLA).

Cardiovascular diseases pose a significant public health risk, and the use of certain lipids has proved good for health. For decades, *n*−3 derived from marine resources (fish oil) has been extensively referenced in cardiology guidelines [70,71,72]. Diets rich in *n*−3 and *n*−6 have shown effective benefits, including long-term protection against most cardiovascular events and associated clinical complications [30,73,74]. To understand the health benefits and assess the nutritional quality of the cyanobacteria in this study, the AI, TI, and h/H values were calculated (Table 1). The AI and TI indices are commonly used to evaluate the potential of the matrix, with lower indices indicating greater benefits due to their association with reduced prevalence of cardiovascular diseases [75]. These indices have previously been used to assess the benefits of microalgae, seaweeds, and fish [76,77,78]. The results for *Laspinema* sp. LEGE 06078 and *Sphaerospermopsis* sp. LEGE 00249 showed high AI values (0.9% and 1.0%, respectively) when compared to marine animals, such as mussels (0.4%), shrimp (0.6%), and octopus (0.7%). In contrast, *Rivulariaceae cyanobacterium* LEGE 06114 exhibited a similar value (0.6%) compared to these marine animals [79,80]. When compared to microalgae and cyanobacteria commercially approved for human consumption in the EU, *Rivulariaceae cyanobacterium* LEGE 06114 had a value similar to that of *Spirulina* sp. (0.7%), but higher when compared to that of *Chlorella vulgaris* (0.2%), *Scenedesmus obliquus* (0.2%), and *Tetraselmis chui* (0.4%), demonstrating good nutritional potential of the lipid extract [26,81].

Lipidomic analysis was performed to disclose the presence of high-value lipids across the cyanobacteria species. Polar lipids, in particular, are considered not only excellent transporters of essential FAs, thus having great nutritional value [35,36,81], but also an excellent source of bioactive compounds with health-promoting properties [26,35]. Considering all the identified lipids across the cyanobacteria, a total of 230 different lipid species were identified, and, curiously, only 26 lipids were common among *Laspinema* sp. LEGE 06078, *Rivulariaceae cyanobacterium* LEGE 06114, and *Sphaerospermopsis* sp. LEGE 00249. This trend shows the unique lipidome of each cyanobacterium indicating a species-specific relation, similarly to what has been previously described for microalgae [36]. The identified lipid species belong to different classes of PLs, GLs, BLs, and NLs. Some of the identified lipid classes were characteristic of one cyanobacteria species; specifically, the GL class of DGGA was only observed in *Rivulariaceae cyanobacterium* LEGE 06114, while the betaine classes of DGTS and MGTS were quite numerous in *Sphaerospermopsis* sp. LEGE 00249. BLs were absent from the cyanobacterium species *Laspinema* sp. LEGE 06078. Between the cyanobacteria, the lipidomes of *Rivulariaceae cyanobacterium* LEGE 06114 and *Laspinema* sp. LEGE 06078 consisted mostly of GL species, while *Sphaerospermopsis* sp. LEGE 00249 consisted mostly of PLs and betaine lipids.

Regarding the GL profile of each cyanobacterium, our results indicate that *Rivulariaceae cyanobacterium* LEGE 06114 and *Laspinema* sp. LEGE 06078 have the highest number of GL species, 48 and 57, respectively, while *Sphaerospermopsis* sp. LEGE 00249 had the lowest number of 28 GLs identified in the present study. The GL species identified for each cyanobacterium belonged to the classes of MGDG, DGDG, and SQDG, while the class of DGGA was only observed in *Rivulariaceae cyanobacterium* LEG 06114. Glycolipids, in general, are constituents of the chloroplast membranes and allow proper function and stabilization of the photosynthetic apparatus [82]. The class of DGGA is less reported in cyanobacteria, although a few studies on microalgae have reported the production of this GL class by SQDG under phosphate-deprived conditions, while paralleling an increase in SQDG species [83,84]. Some GLs identified across all cyanobacteria have been previously described with intrinsic bioactive properties, namely anti-inflammatory, anti-viral, and anti-obesity [85,86,87]. *Rivulariaceae cyanobacterium* LEGE 06114, with its distinctive DGGA profile, may offer advantages in developing skincare products or bioactive ingredients with anti-inflammatory properties. GL species MGDG 16:0_18:3, MGDG 16:0_18:4, and SQDG 16:0_18:3 inhibited nitric oxide production by reducing the expression of inducible nitric oxide synthase (iNOS) in macrophages activated with lipopolysaccharide (LPS), the pro-inflammatory stimulus [86,88]. Other activities include the anti-viral potential of MGDG species isolated from the cyanobacteria *Oscillatoria raoi*, *O. trichoides*, *O. limnetica*, and *Phormidium tenue*, which inhibited reverse transcriptase from HIV-1 [85]. The anti-obesity properties of GL were observed for MGDG 16:0_18:2, which inhibited TG accumulation in 3T3-L1 adipocytes [87]. The presence of bioactive GL underscores the potential of these cyanobacteria as potential sources of high-value compounds.

PLs were particularly abundant in *Sphaerospermopsis* sp. LEGE 00249 and *Laspinema* sp. LEGE 06078, while only 10 PL species were identified in *Rivulariaceae cyanobacterium* LEGE 06114. Each species exhibited a distinct PL profile. Notably, *Sphaerospermopsis* sp. LEGE 00249 had a higher number of PC species, highlighting its diverse and enriched structural PL content. In contrast, *Laspinema* sp. LEGE 06078 was characterized by an elevated number of PG species (18), a key component of chloroplast thylakoid membranes [82]. This cyanobacterium exhibited a higher concentration of GLs, which are exclusive to chloroplasts, supporting the evolutionary link between cyanobacteria and chloroplasts, as cyanobacteria are widely regarded as the ancestors of chloroplasts [89,90]. PLs are considered excellent carriers of FAs as they have higher bioavailability when esterified to PLs compared to their free forms [35]. Their functional properties, including emulsifying and surfactant capabilities, make them valuable for biotechnological applications, such as lecithin production for food and as neutral bases for cosmetic formulations [91,92]. Furthermore, PLs possess intrinsic bioactive properties, particularly anti-inflammatory activity. For example, lysophosphatidylcholine (LPC 16:0), which was identified in this work in *Sphaerospermopsis* sp. LEGE 00249, has been shown to inhibit Tumor necrosis factor (TNF-α) production induced by LPS in Tohoku Hospital Pediatrics-1 (THP-1) human monocytes [93].

TGs were particularly abundant in *Laspinema* sp. LEGE 06078 and *Rivulariaceae cyanobacterium* LEGE 06114, containing 38 and 32 species, respectively. *Sphaerospermopsis* sp. LEGE 00249 showed fewer TG species (12), which may be associated with different metabolic focuses or storage strategies, as this species has more PLs compared to the other two cyanobacteria in this study. TGs are the primary form of energy storage in organisms, and these variations in TG profiles may reflect differences in energy storage capacities and the role of these lipids in stress responses or environmental adaptation. A study on nutrient deprivation-induced TG accumulation in microalgae [94] highlighted the widespread biotechnological interest in the production of neutral lipids from microalgae [95]. DGs serve as key intermediates in the synthesis of phospholipids and glycerolipids such as triacylglycerols. They can also function in cellular signaling, with their physical properties influencing cell membrane biophysics and acting as important immunomodulators [96].

However, differences in the FA and lipidome profile were observed, particularly between *Sphaerospermopsis* sp. LEGE 00249 and the other two cyanobacterial strains. Interestingly, the FA profiles of *Laspinema* sp. LEGE 06078 and *Rivulariaceae cyanobacterium* LEGE 06114 were relatively similar (Supplementary Figure S6), but one is from freshwater and the other, marine. Interestingly, these environmental differences between the two cyanobacteria were not reflected in the FA profile. However, when we analyzed the lipidome of each individual species, their profile was completely distinct, suggesting that cyanobacteria have their own lipid signature.

Some of the identified lipid species have been reported with different properties, such as antitumoral and anti-inflammatory activities, such as in the case of glycolipids [97]. Several studies have reported antioxidant activities in lipid extracts from spirulina [26,32]. Therefore, we investigated the antioxidant potential of lipid extracts from thestudied cyanobacterial strains. Antioxidant compounds are crucial for combating oxidative stress and contribute to the prevention of cellular damage and chronic diseases, in addition to having potential biotechnological and pharmaceuticals applications [98,99]. These antioxidant compounds also play a protective role against food spoilage, as reported in studies with seaweed [100]. Antioxidant compounds present in cyanobacteria extracts can neutralize the free radicals, preventing premature aging and other organs damage, as demonstrated in studies by Conde et al. [26] and Silva et al. [101]. Another characteristic is the presence of antioxidant lipids, such as PUFAs, which scavenge oxidative radicals and prevent damage caused by oxidation [102]. In our study, we employed ABTS^•+^ and DPPH^•^ assays to assess the antioxidant properties of cyanobacteria lipid extracts.

The lipid extracts of the cyanobacteria demonstrated the ability to inhibit 50% of the ABTS^•+^ radical at the tested concentrations, as shown in Figure 6, indicating the presence of compounds with antioxidant properties. The lowest IC50 value was observed for *Laspinema* sp. LEGE 06078 and *Rivulariaceae cyanobacterium* LEGE 06114, indicating that this species also possesses antioxidant capacity. *Sphaerospermopsis* sp. LEGE 00249 showed the lowest antioxidant activity compared to the other two cyanobacteria. Thus, the lipid profile is critical for understanding the antioxidant performance, since *Laspinema* sp. LEGE 06078 and *Rivulariaceae cyanobacterium* LEGE 06114 presented more PUFAs when compared with *Sphaerospermopsis* sp. LEGE 00249. Conde et al. [26] conducted a study with six microalgae and one cyanobacterium commercially approved for human consumption in the EU. These results were similar to the ones obtained in our study. The *S. obliquus* showed the lowest IC50 (29.4%), and *N. oceanica*, the highest IC50 (101.9%), while *Spirulina* sp. had IC50 38.7% [26]. Our results highlight *Laspinema* sp. LEGE 06078 and *Rivulariaceae cyanobacterium* LEGE 06114 as potential candidates for biotechnological applications aimed at the production of natural antioxidants. The DPPH^•^ assay showed a 30% radical inhibition for all lipid extracts, with a trend similar to that observed in the ABTS^•+^ assay. Our DPPH assay results demonstrate better results when compared to the ethanolic extracts (IC25) of *Synechocystis salina* LEGE 06099 (482.0 μg mL^−1^) and *Nodosilinea nodulosa* LEGE 06102 (764.1 μg mL^−1^) in a study by Morone et al. [98]. Another work conducted by Pan-utai et al. [103] showed that microencapsulated C-PC from *L. platensis* (formerly *A. platensis*) has high antioxidant capacity and is effective in neutralizing free radicals (IC50 ranging from 7.6 to 13.5 mg mL^−1^). The compounds present in cyanobacterial lipid extracts could reduce the intracellular oxidative stress, which is a key factor. These compounds also play important roles in the adaptative response to oxidative stress through constant exposure to UV radiation [104]. Between the main properties of photosynthetic organisms and human needs, the antioxidant potential of their extracts plays a crucial role, since living cells can generate free radicals as products of physiological and biochemical processes. These results imply that cyanobacteria strain, namely *Laspinema* sp. LEGE 06078 and *Rivulariaceae cyanobacterium* LEGE 06114, with a similar chemotaxonomy linkage distance (Supplementary Figure S6), can be suggested for cosmeceutical application due to their potential to provide antiaging effects.

Lipid-lowering in zebrafish larvae is commonly used as a model for metabolic diseases, such as obesity, due to the functional conservation in lipid metabolism and adipose biology, among other applications [105]. In our study, the lipid-lowering capacity of cyanobacterial lipid extracts from *Sphaerospermopsis* sp. LEGE 00249 reduced significantly to about 41% of Nile red staining (Figure 7) compared with REV (a polyphenol of the class of stilbenes, described to inhibit neuropeptide Y and FA synthase of activation). In other studies, the lipid-lowering was identified in methanolic extracts of *Cyanobium* sp. BACA0019 (39.1%), *Pseudocalidococcus azoricus* BACA0433 (40.4%), and *Pegethrix atlantica* BACA0077 (50.7%) [106]. Furthermore, fractions of 15 cyanobacterial strains exhibited lipid-lowering above 50%, with the majority from marine ecosystems [107].

In this context, cyanobacterial lipid extracts as potential “anti-obesity” agents have been the subject of increasing scientific interest [108,109]. Since 1981, spirulina has been speculated to have lipid-lowering capabilities [44]. Spirulina was reported for its ability to promote weight loss and improve blood lipid levels [110], as well as to demonstrate beneficial cardiovascular effects even with short-term, low-dose spirulina supplementation [30,111], along with literature reviews on its use for such purposes [112]. However, controversies about the beneficial effects of spirulina have been reported in the scientific literature [113]. The bioactivities observed in cyanobacterial lipid extracts of this work, namely the lipid-lowering capacity, are related to their specific lipid composition, with different levels of FAs and PUFAs, as an example. PUFAs have been described as modulators of lipid metabolism by activating the peroxisome proliferator-activated receptor (PPARs), and enhancing β-oxidation, leading to a decrease in lipid accumulation [114]. Another important point is that *Sphaerospermopsis* sp. LEGE 00249 presented a higher number of betaine lipids when compared with the other two cyanobacteria strains. In microalgae, DGTS serves as a substitute for phospholipids under phosphate-limited conditions and is also involved in stress adaptation processes [115]. Also, DGTS is rich in EPA and other omega-3 PUFA, providing beneficial properties such as anti-inflammatory activity [115]. In zebrafish models, dietary betaine supplementations have been shown to regulate fatty acid synthesis [116]. Since direct studies on the lipid-lowering effects of betaine lipids in zebrafish are limited, further research is needed to elucidate the specific mechanism of betaine lipids to influence lipid homeostasis in other organisms.

Given this scenario, there is an urgent need to develop effective approaches, such as new natural therapies, to prevent and treat obesity, including the search for novel compounds that can regulate lipid metabolism, reduce fat accumulation, and improve overall health. Several polar compounds including lipids are already associated with beneficial effects for obesity. Some chlorophyll derivatives such as hydroxypheophytine a and hydroxyfarnesyl have been isolated from marine cyanobacteria due to their strong lipid-lowering activity [44]. The fat-soluble vitamin *K* family was reported to reduce neutral lipids in zebrafish larvae with interesting differences regarding potency and biological effects between their isoforms [117]. The lipid extract of the wild plant species *Sonchus oleraceus* inhibited adipogenesis and attenuated the high-fat-diet-induced obesity in mice following a treatment at 0.3 mg g^−1^ for a month; however, the active compound was not yet identified [118]. Polar lipids naturally present in milk, with a high content of sphingomyelin, were shown to reduce high-fat-diet-induced body weight gain and alter the microbiota composition [119]. In our own data, lipid-lowering was observed for a lipidic extract of the strain of *Sphaerospermopsis* sp. LEGE 00249, and future work should focus on isolating the responsible lipid species.

## 4. Materials and Methods

### 4.1. Reagents

For biomass production, deionized water was used for the preparation of the culture medium (LabTower EDI 15, Thermo Electron LED, Niederelbert, Germany). For the preparation of the Z8 medium, different reagents were used according to Kotai [120], and as available at https://lege.ciimar.up.pt/knowledgebase/ (accessed on 10 February 2025). For lipid analysis, HPLC-grade methanol (MeOH), absolute ethanol, hexane, acetonitrile, isopropanol, and dichloromethane (CH_2_Cl_2_) were used as solvents (Fisher Scientific Ltd., Loughborough, UK). Lipid internal standards were purchased from Avanti Polar Lipids, Inc. (Alabaster, AL, USA), as described by Couto et al. [36]. All the other reagents were purchased from major commercial sources and were of the highest grade of purity available. Milli-Q water (Synergy, Millipore Corporation, Billerica, MA, USA) was used. Furthermore, 2,2-diphenyl-1-picrylhydrazy radical (DPPH^•^) was purchased from Aldrich (Milwaukee, WI), 2,2′-Azino-bis (3-ethylbenz othiazoline-6-sulfonic acid) diammonium salt (ABTS^•+^) was obtained from Fluka (Buchs, Switzerland), and 6-hydroxy-2,5,7,8-tetramethylchromane-2-carboxylic acid (Trolox) was purchased from Sigma-Aldrich (St Louis, MO, USA) [26]. The reagents used for the lipid-lowering assay are already reported by Urbatzka et al. [121].

### 4.2. Cyanobacterial Material

#### 4.2.1. Strains and Biomass Production

**Figure 8:**
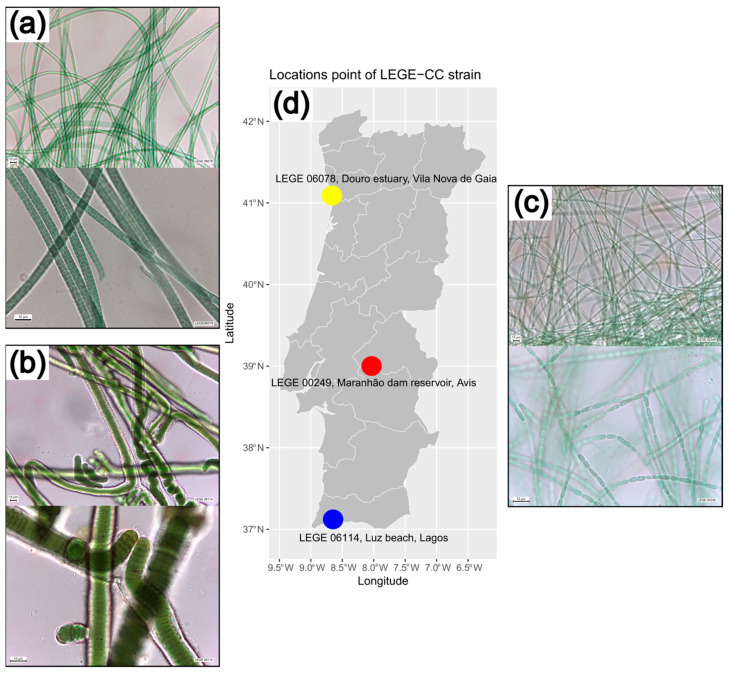
Microphotographs and sampling point of cyanobacterial strains. (**a**) Magnification ranged from 400 to 1000× of *Laspinema* sp. LEGE 06078; (**b**) magnification ranged from 400 to 1000× of *Rivulariaceae cyanobacterium* LEGE 06114; (**c**) magnification ranged from 400 to 1000× of *Sphaerospermopsis* sp. LEGE 00249; (**d**) map showing the cyanobacteria sampling sites in Portugal. Different colors indicate cities where samples were collected.

These strains were collected from different regions of Portugal (Figure 8) [45] and provided by the Blue Biotechnology and Ecotoxicology Culture Collection (LEGE-CC). For *Laspinema* sp. LEGE 06078 and *Sphaerospermopsis* sp. LEGE 00249, Z8 culture medium was used, while for *Rivulariaceae cyanobacterium* LEGE 06114, Z8 culture medium was used plus 25 g L^−1^ of tropical marine salt (salt Tropical Marin, Berlin, Germany) and + 1‰ of vitamin B12 (Z8_25_) [120,122,123]. The culture media were prepared using deionized and autoclaved water. To obtain the biomass, firstly, the strains were cultivated in 40 mL flasks for 2 weeks (without aeration). After this, the cultures were transferred for a new culture media (800 mL) with continuous aeration for 4 weeks. Cultures were kept under controlled temperature conditions (21 °C ± 2 °C), with a photoperiod of 16/8 h light/dark cycle (L/D) and light intensity of 20 µmol m^−2^ s^−1^ (AsenseTek Lighting Passport, Biosystems, United Kingdom) with fluorescent light (Acardia, United Kingdom). In the next step, biomass harvest was carried by centrifugation (Heraeus Megafuge 16R, Thermo Scientific, Osterode am Harz, Germany) at 6000× *g* for 10 min at room temperature. For the Z8_25_ culture medium, an extra step was added, consisting of three washes with deionized water to remove the salts. The samples were dried in a freeze dryer (Lyoquest-55, Telstar Technologies, S. L., Terrassa, Spain) and stored away from light. Finally, the samples were stored at −20 °C.

#### 4.2.2. DNA Extraction, PCR Amplification, and Sequencing

To remove bacteria and marine salts (present in marine strain), the cyanobacteria cells were harvested from the cultures and centrifuged at 12,000× *g* for 3 min at room temperature (Micro Star 17R, VWR, Radnor, PA, USA). Subsequently the supernatant was removed, and fresh Z8 medium was added for the next centrifugation. This process was repeated three times to remove bacteria and marine salts. The total genomic DNA (gDNA) of the strains was extracted using the PureLink Genomic DNA Mini kit (Invitrogen, Waltham, MA, USA), following the manufacturer’s instructions provided for Gram-negative bacteria. Specific cyanobacteria primers were used for gene amplification, including primers 27SF [124] and 23SR [125]. The PCR reactions, purification of PCR products, and sequencing were performed according to Oliveira et al. [8]. To assess the presence and quality of the DNA obtained from the extraction and PCR, we performed electrophoresis on, respectively, 1% (*w*/*v*) and 1.5% (*w*/*v*) agarose gel stained with SYBR Safe DNA gel stain (Invitrogen by Thermo Fisher Scientific, Waltham, MA, USA). The confirmation of high-molecular-weight DNA was based on the presence of clear bands observed in the gel.

The sequencing was performed via sanger dideoxy sequencing at GATC Biotech (Ebersberg, Germany), and the nucleotide sequences obtained were manually inspected for quality and assembled using the Geneious Prime 2023.2.1 software (Biomatters Ltd., Auckland, New Zealand). The obtained sequences were checked for possible chimera formation using the DECIPHER software 2.27.2 [126] and were deposited in the GenBank (National Center for Biotechnology Information, NCBI). Their GenBank IDs are exhibited in the phylogenetic trees.

#### 4.2.3. Potential for Cyanotoxin Production

The potential for cyanotoxin production was determined using molecular biology methods on the gDNA to check whether the strains are toxin producers. PCR was used to amplify toxin synthesis-related genes encoding the amino transferase (AMT) of the microcystin and nodularin synthetase complexes (*mcyE*), saxitoxin (*sxtI*), anatoxin (*anaC*), and cylindrospermopsin (*cyrJ*) using specific primer sets [127,128,129,130], and PCR programs described previously by Benredjem et al. [131] (Supplementary Table S4). PCR validation of the amplified samples was obtained using a positive control strain of *Microcystis aeruginosa* LEGE 00063 for *mcyE*, *Aphanizomenon gracile* LMECYA 040 for *sxtI*, *Anabaena* sp. LEGE X-002 for *anaC*, and *Cylindrospermopsis raciborskii* LEGE 97047 for *cyrJ*. PCR products performed according to Oliveira et al. [8]. To assess the presence and quality of the DNA obtained from extraction and PCR, we performed electrophoresis on, respectively, 1% (*w*/*v*) and 1.5% (*w*/*v*) agarose gel stained with SYBR Safe DNA gel stain (Invitrogen by Thermo Fisher Scientific, Waltham, MA, USA). The confirmation of high-molecular-weight DNA was based on the presence of clear bands observed in the gel.

#### 4.2.4. Phylogenetic Analysis

The 16S rRNA sequences of *Laspinema* sp. LEGE 06078, *Rivulariaceae cyanobacterium* LEGE 06114, and *Sphaerospermopsis* sp. LEGE 00249 were aligned with the Oscillatoriales and Nostocales reference strains’ sequences using the ClustalW algorithm [132]. The phylogeny was inferred using the Maximum Likelihood (ML) method [133], in MEGA 11 (bootstrap = 1000) [134], based on the General Time Reversible model [135], which was the nucleotide substitution model that best fit the alignment data as evaluated by the corrected Akaike Information Criterion [136]. A discrete Gamma distribution (+G) was used to model the evolutionary rate differences among sites, while the rate variation model allowed some sites to be evolutionarily invariable (+I). The final phylogenetic tree was edited on Interactive Tree of Life (iTOL) [137] and Inkscape 1.2 [138]. The similarity values (*p*-distance) were calculated using MEGA11. For the identification of *Laspinema* sp. LEGE 06078, *Rivulariaceae cyanobacterium* LEGE 06114, and *Sphaerospermopsis* sp. LEGE 00249, values higher than 95% of 16S rRNA similarity (*p*-distance) with any cyanobacterial strain were indicative to belong to the same genus [139,140,141].

#### 4.2.5. Morphological Analysis

The strains were microphotographed and analyzed using LEICA LAS version 4.12.0 image analysis software (Leica Microsystems Ltd., Wetzlar, Germany, and CMS GmbH, Switzerland). The form and dimensions of vegetative cells (both intercalary and terminal), the quantity of trichomes per filament, and the existence or absence of sheaths, constrictions at the cross-wall, and necridic cells were documented. To visualize the mucilaginous envelopes, China Ink was used for staining. The measurements were performed for each characteristic of the strain (20 to 30 measurements) and were carried out at various positions of the slide preparation.

### 4.3. Biomass Proximate Composition

The entire process was executed at Greencolab (https://www.greencolab.com/ (accessed on 11 October 2024)). The proximate composition analysis of biomass was conducted for the cyanobacterial strains by estimating the contents of ash, protein, and carbohydrates (estimated using the difference in total biomass weight). Dried samples were ground to powder on a ball mill (Retsch MM 300, Retsch GmbH, Haan, Germany) with tungsten beads (3 mm). For the quantification of total nitrogen, hydrogen, and carbon, approximately 1 mg (*n* = 3) was used, in a CHN elemental analyzer (Vario EL, Elemental Analyzer system, GmbH, Hanau, Germany). Protein content was estimated according to Dumas’ method (ISO 16634/AOAC 990.03), by multiplying total nitrogen by a conversion factor of 6.25, according to FAO [142]. The carbohydrate contents were only estimated. The ash content was determined by the complete combustion of the freeze-dried biomass, approximately 30 mg (*n* = 3) in a furnace (J. P. Selecta, Sel horn R9-L, Barcelona, Spain) at 550 °C for 6 h [143].

### 4.4. Lipid Extraction Procedure

Lipid extraction from cyanobacteria cultures was carried out based on the Folch method [26,144]. Firstly, 2.0 mL of CH_2_Cl_2_:MeOH (2:1, *v*/*v*) in a glass tube was added to 25 mg of lyophilized biomass (*n* = 5) per cyanobacteria, and vortexed for 2 min. After centrifugation (Selecta JP Mixtasel, Abrera, Barcelona, Spain) at 670× *g* for 10 min, the organic phase was collected in a new glass tube. This process was repeated three more times, and the combined organic phases were then dried under nitrogen gas. This extract was redissolved in 2 mL of CH_2_Cl_2_, and 1 mL of MeOH, followed by 1 min of vortexing. Then, 0.75 mL of Mili-Q water was added and vortexed for 2 min, followed by centrifugation at 670× *g* for 10 min. The organic phase was collected in a new glass tube and the aqueous phase was reextracted with 2 mL of CH_2_Cl_2_ two more times as mentioned above. The combined organic phases were dried under nitrogen gas, and the extract was transferred to pre-weighed amber vials, and subsequently dried, weighed, and stored at −20 °C. Lipid content, expressed as a percentage of dry weight (DW), was determined using the following equation (Equation (1)), with the results of five replicates presented as the mean ± standard deviation:

(1) $$\mathrm{Lipid}\;\mathrm{content}\;\mathrm{yield}\left(\%\;\mathrm{DW}\frac{W}{W}\right)=\frac{\mathrm{Weight}\;\mathrm{of}\;\mathrm{the}\;\mathrm{lipid}\;\mathrm{extract}\;(g)}{\mathrm{Weigth}\;\mathrm{of}\;\mathrm{biomass}\;(g)}\;\times \;100$$

#### 4.4.1. Fatty Acid Analysis by Gas Chromatography–Mass Spectrometry (GC–MS)

##### Data Acquisition

The FA profile of the different cyanobacteria was determined by GC–MS. FA methyl esters were prepared by alkaline transmethylation reaction using a methanolic solution of potassium hydroxide (2.0 M) as previously described [26]. A volume of 2.0 μL of FAMEs, containing 1.0 μg mL^−1^ of methyl nonadecanoate (internal standard), was injected into a GC instrument (Agilent Technologies 8860 GC System, Santa Clara, CA, USA) equipped with a DB-FFAP column with the following specifications: 30 m long, 0.32 mm internal diameter, and 0.25 μm film thickness (J&W Scientific, Folsom, CA, USA). This was connected to an Agilent 5977B Mass Selective Detector (Agilent Technologies Inc., Santa Clara, CA, USA) operating with electron impact ionization at 70 eV and a scanning range of *m*/*z* 50–550 (1 s cycle in full-scan mode). The following running conditions were employed: helium as carrier gas (constant flow of 1.4 mL min^−1^), inlet temperature of 220 °C, detector temperature of 230 °C, and injection volume of 2 μL (splitless). The oven temperature was programmed as follows: 58 °C for 2 min, 25 °C min^−1^ to 160 °C, 2 °C min^−1^ to 210 °C, and 30 °C min^−1^ to 225 °C (held for 20 min). The data acquisition software used was GC–MS 5977B/Enhanced MassHunter (Agilent Technologies Inc., Santa Clara, CA, USA).

##### Data Analysis

FA assignment involved identification through retention time comparison with those of a commercial standard mixture (Sigma-Aldrich, St. Louis, MO, USA) and 1.0 µg mL^−1^ of methyl nonadecanoate (Sigma-Aldrich, St. Louis, MO, USA). Five independent replicates were injected (*n* = 5). Mass spectrum analysis was also used for identification by checking the typical peaks present in the mass spectra of these FAs and their abundances, and comparisons with the mass spectra obtained for the standard mixture. The NIST chemical database library (https://www.nist.gov/srd/nist-standard-reference-databases-analytical-chemistry (accessed on 1 February 2025)) and LipidMAP database (https://www.lipidmaps.org/ (accessed on 3 February 2025)) were also used for the identification of lipids. FA peaks were integrated using the software Agilent MassHunter Qualitative Analysis 10.0. FA quantification was performed using calibration curves for each FA. Results were expressed as the amount of FAs in biomass (mg FA g^−1^). The AI, TI, and h/H indices were calculated using the following equations (Equations (2)–(4)), as proposed by Ulbricht and Southgate [145]:

(2) $$\mathrm{AI}\;=\frac{\left[\mathrm{FA}\;12:0+(4\;\times \;\mathrm{FA}\;14:0)+\mathrm{FA}\;16:0\right]}{[\sum \mathrm{MUFA}+\sum \left(n-6\right)+\sum (n-3)]}$$

(3) $$\mathrm{TI}\;=\frac{\left[\mathrm{FA}\;14:0+\mathrm{FA}\;16:0+\mathrm{FA}\;18:0\right]}{[\left(0.5\;\times \;\mathrm{FA}\;18:1\right)+(0.5\;\times \;\sum \mathrm{Other}\;\mathrm{MUFA})+(0.5\;\times \;\sum \left(n-6)\right)+(3\;\times \;\sum \left(n-3)\right)+(\frac{\sum \left(n-3\right)}{\sum \left(n-6\right)})]}$$

(4) $$(h/H)\;=\frac{\left[\mathrm{FA}\;18:1\;\left(n-9\right)+\mathrm{FA}\;18:2\;\left(n-6\right)+\mathrm{FA}\;18:3\;\left(n-3\right)+\mathrm{FA}\;20:4\;\left(n-6\right)+\mathrm{FA}\;20:5\;\left(n-3\right)\right]}{\left[\mathrm{FA}\;14:0+\mathrm{FA}\;16:0\right]}$$

### 4.5. Lipidomic Analysis

#### 4.5.1. Data Acquisition

The lipid extracts of the different cyanobacteria were analyzed by reverse phase liquid chromatography in an Ultimate 3000 Dionex (Thermo Fisher Scientific, Bremen, Germany) using an Ascentis^®^ Express C18 column 2.1 × 150 mm, 2.7 μm, (Merck KGaA, Darmstadt, Germany) coupled to the Q-Exactive^®^ hybrid quadrupole Orbitrap mass spectrometer (Thermo Fisher, Scientific, Bremen, Germany). Mobile phase A comprised water/acetonitrile (40/60%) with 10 mM ammonium formate and 0.1% formic acid. Mobile phase B was composed of isopropanol/acetonitrile (90/10%) with 10 mM ammonium formate and 0.1% formic acid. The following gradient was applied: 32% B at 0 min, 45% B at 1.5 min, 52% B at 4 min, 58% B at 5 min, 66% B at 8 min, 70% B at 11 min, 85% B at 14 min, 97% B at 18 min, 97% B at 25 min, 32% B at 25.01 min, and 32% B at 33 min.

A mixture of 30 μg of lipid extract (in 10 μL of dichloromethane), 82 μL of a solvent system consisting of 50% isopropanol/50% methanol, and 8 μL of lipid standards mixture (dMPC—0.04 μg, dMPE—0.04 μg, LPC—0.04 μg, dPPI—0.08 μg, CL (14:0) 4–0.16 μg; dMPG—0.024 μg, Cer(17:0/d18:1)—0.08 μg) was prepared for each mixture, and 5 μL was loaded into the column at 50 °C with a flow-rate of 260 μL min^−1^. The mass spectrometer operated in simultaneous positive (ESI 3.0 kV) and negative (ESI −2.7 kV) modes. The capillary temperature was 320 °C, and the sheath gas flow was 35 U. Data were acquired in full-scan mode with a high resolution of 70,000, automatic gain control (AGC) target of 3 × 10^6^, in an *m*/*z* range of 300–1600, 2 microscans, and maximum inject time (IT) of 100 ms. The tandem mass spectra (MS/MS) were obtained with a resolution of 17,500, AGC target of 1 × 10^5^, 1 microscan, and maximum IT of 100 ms. The cycles consisted of one full-scan mass spectrum and ten data-dependent MS/MS scans, which were repeated continuously throughout the experiments with a dynamic exclusion of 30 s and intensity threshold of 8 × 10^4^. Normalized collision energy TM (CE) ranged among 20, 24, and 28 eV in the negative mode and between 25 and 30 eV in the positive mode. Data acquisition was performed using the Xcalibur data system (V3.3, Thermo Fisher Scientific, Bremen, Germany).

#### 4.5.2. Data Analysis

Lipid identification was performed based on retention time, mass accuracy observed in LC-MS spectra, and LC-MS/MS spectrum interpretation. MSDial 4.6 software was used for peak detection [146], compound identification, and the generation of a list of identified species. The generated template was further used in Mzmine 2.53 software [147]. This software was used for filtering LC-MS raw data, peak detection, peak processing, and assignment against the template generated with MSDial. Only peaks within 5 ppm of the lipid exact mass and intensity higher than 1 × 10^5^ were considered. Relative quantification was performed by normalizing the peak areas of the extracted ion chromatograms (EICs) with the peak areas of the internal standards. The typical fragmentation rules used to generate the assignments are described in Supplementary Table S5, and are in accordance with the literature, as previously described by Rey et al. [35].

### 4.6. Antioxidant Scavenging Activity

#### 4.6.1. ABTS Cation Radical Scavenging Assay

To evaluate the antioxidant scavenging activity against the ABTS^•+^, we used a method previously described with some modifications [148,149]. Briefly, 150 µL of an ethanolic dilution of the extracts (25, 125, 250, 500 µg mL^−1^) or 150 µL of the Trolox standard solution (5.0, 12.5, 25.0, 37.5 µmol L^−1^ in ethanol) was mixed in triplicate with 150 µL of an ABTS^•+^ working solution in ethanol (absorbance ≈ 0.9, 734 nm, absorbance decrease < 10%). Then, the mixture was incubated for 120 min, and the absorbance was measured at 734 nm every 5 min (Multiskan GO 1.00.38, Thermo Scientific, Hudson, NH, USA). A control of each sample was prepared by replacing the ABTS^•+^ solution with ethanol. The antioxidant activity, expressed as a percentage of the inhibition of the ABTS radical, was calculated using Equation (5). The concentration of samples able to scavenge 50% of ABTS radical (IC50) after 120 min of reaction was calculated via linear regression, plotting the concentration of lipid extract versus the percentage of the inhibition curve. The activity is expressed in TEs, which were calculated using Equation (6), where IC50 values are the concentration of sample or of Trolox that induces the reduction in the ABTS^•+^ radical to 50%. All measurements were performed in triplicate (*n* = 3).

(5) $$\mathrm{Inhibition}\;\%\;=\frac{\left({\mathrm{Abs}}_{{\mathrm{ABTS}}^{+•}}-{\mathrm{Abs}}_{\mathrm{Sample}-\mathrm{control}}\right)}{{\mathrm{Abs}}_{{\mathrm{ABTS}}^{+•}}}\;\times \;100$$

(6) $$\mathrm{TE}=\frac{{\mathrm{IC}}_{50}\;\mathrm{Trolox}\;(µ\mathrm{mol}/g)}{{\mathrm{IC}}_{50}\;\mathrm{of}\;\mathrm{samples}\;(µg/\mathrm{mL})}\;\times \;1000$$

#### 4.6.2. DPPH Radical Scavenging Assay

To evaluate the antioxidant scavenging activity against the 2,2-diphenyl-1-picrylhydrazyl radical (DPPH^•^), we used a method previously described [148,150], with some modifications. In the first step, 150 µL of an ethanolic dilution of the extracts (25, 125, 250, 500 µg mL^−1^) or 150 µL of the Trolox standard solution (5.0, 12.5, 25.0, 37.5 µmol L^−1^ in ethanol) was mixed in triplicate with 150 µL of a DPPH^•^ working solution in ethanol (absorbance ≈ 0.9, 517 nm, absorbance decrease < 10%). Then, the mixture was incubated for 120 min, and the absorbance was measured at 517 nm every 5 min (Multiskan GO 1.00.38, Thermo Scientific, Hudson, NH, USA). A control of each sample was prepared by replacing the DPPH^•^ solution with ethanol. The antioxidant activity, expressed as a percentage of the inhibition of the DPPH radical, was calculated using the following equations (Equations (5) and (6)) (AbsABTS^•+^ substituted by AbsDPPH^•^) and expressed in IC30 and TEs, where IC30 values are the concentration of sample or of Trolox that induces the reduction in the DPPH^•^ radical to 30%. All measurements were performed in triplicate (*n* = 3).

### 4.7. Lipid-Lowering Assay

To characterize the lipid-lowering activity of the lipid extracts from the cyanobacteria, the zebrafish Nile red fat metabolism assay was used by measuring fluorescence intensity (MFI). Adult Danio rerio were maintained in appropriate conditions in an aquaculture system, involving the control of temperature, light, and water quality as described by Urbatzka et al. [121]. Approval by an ethics committee was not necessary to carry out the bioassays, as the chosen procedures are not considered animal experimentation according to EC Directive 86/609/EEC for animal experimentation. After reproduction (according to the guideline available at https://zfin.org/ (accessed on 15 February 2025)), the eggs were collected and observed under a magnifying glass for selection (Supplementary Figure S8). The eggs were kept in an incubator at 28 °C in specific medium for zebrafish embryos—E3 (NaCl; KCl; CaCl_2_·2H_2_O and MgCl_2_·6H_2_O) with methylene blue—MB (Sigma-Aldrich). One day after fertilization (DPF), 1-phenyl-2-thiourea (PTU) was added at 200 µM to inhibit pigmentation. For the entire process, we followed protocol No. 236: Fish Embryo Acute Toxicity (FET) Test, from the Organization for Economic Co-operation and Development (OECD). To carry out the bioassay, zebrafish larvae at 3 DPF were used. Previously reported anti-obesity biological activity assays were used to evaluate lipid-lowering activity, using the Nile red fat metabolism assay in zebrafish [121,151]. From 3 to 5 DPF, zebrafish larvae were exposed to the different lipid extracts (see the Lipid Extraction Procedure section) in a 24-well plate with a density of 6 larvae/well per cyanobacterial lipid extract, in quintuplet (*n* = 30) in two independent experiments (separated by 15 days). A positive solvent control (1% DMSO) and a negative control (REV, resveratrol, 50 µM) were included in the experiments. The zebrafish larvae were exposed to a final concentration of 10 µg mL^−1^ for each cyanobacterial lipid extract. Eighteen hours before reading, the neutral lipids were stained with 10 ng mL^−1^ Nile red per well. At 5 DPF, zebrafish were anesthetized (tricaine MS-222, 0.03%) for imaging. Fluorescence was analyzed with an automated microscope (Thunder Imaging Systems, Leica Microsystems CMS GmbH, Wetzlar, Germany). Fluorescence intensity was quantified individually in zebrafish larvae with ImageJ version 1.53 (https://imagej.net/ij/index.html (accessed on 3 March 2025)).

### 4.8. Statistical Analysis

For analysis, various R packages were employed to ensure robust and accurate data interpretation. Data were first checked for normality using the Shapiro–Wilk test, implemented through the ‘shapiro.test()’ function from the ‘stats’ package. Homogeneity of variances was assessed using Levene’s test, which was conducted using the ‘leveneTest()’ function from the ‘car’ package. For comparisons between groups, a linear model (one-way ANOVA) was adjusted using the ‘lm()’ function, followed by post hoc Tukey’s HSD tests, applied using the ‘TukeyHSD()’ function to identify significant differences between specific groups. These functions are inside the ‘stats’ package. All statistical tests were conducted with a significance level set at *p* < 0.05. Data visualization and the generation of summary statistics were performed using the ‘ggplot2’ or ‘R base function’ package, which facilitated the creation of barplots, boxplots, and other graphical representations to illustrate the distribution and significance of the data. To assess differences between treatments with few data points (less than 30), we used a non-parametric approach based on bootstrapping with 10,000 iterations (for antioxidant analysis), as this method is especially useful for small samples or samples that do not follow a normal distribution. The empirical *p*-value was calculated as the proportion of resampled mean differences greater than or equal to the observed difference. Non-metric multidimensional scaling, employing the Bray–Curtis distance, was performed with the metaMDS function, from the vegan package. PCA was performed with the PCA function from the FactoMineR package. To perform the ‘chemotaxonomy()’ function of FAs, we used the vegan package. For the analysis of lipidome, we also used the ‘chord diagram()’ function to from the circlize package, and we also used the ‘venn diagram()’ function from the vennDiagram package. The map was created using the geom_sf() function from the ggplot2 package in R. First, the geographic data were loaded and prepared using the sf package. The ggplot() function initiated the plot, and geom_sf() was used to add the spatial data layer to the map [152].

## 5. Conclusions

This study reinforces cyanobacteria as promising sources of bioactive compounds with potential applications in food and biotechnology. We integrated the biochemical, FA, and lipidomic characterization of three cyanobacterial strains: *Laspinema* sp. LEGE 06078, *Rivulariaceae cyanobacterium* LEGE 06114, and *Sphaerospermopsis* sp. LEGE 00249. We revealed the need for a revision of *Rivulariaceae cyanobacterium* LEGE 06114, which may need to be classified in a new genus. Notably, freshwater strains exhibited higher protein content, while marine strains accumulated more carbohydrates, indicating ecological adaptations reflected in their biochemical composition. Lipid content also differed with significant variations in FA composition. *Rivulariaceae cyanobacterium* LEGE 06114 presented a particularly high abundance of PUFAs, reinforcing its nutritional potential as a source of essential *n*–3 and *n*–6 Fas, and is crucial for cardiovascular and metabolic health. The lipidomic profile, conducted for the first time for the cyanobacterial strains from this study, confirmed a diverse species-specific profile, with *Laspinema* sp. LEGE 06078 displaying the highest total lipids content and *Sphaerospermopsis* sp. LEGE 00249 highlighted by the number of BLs. Functionally, lipid extracts from these cyanobacterial strains demonstrated antioxidant potential and lipid-lowering activity in zebrafish larvae. Some of these lipids were associated with bioactivities reported with bioactive activities and have been linked to, namely, anti-inflammatory, anti-viral, and lipid-lowering properties, suggesting applications in functional foods and pharmaceuticals. These findings reinforce cyanobacteria as promising candidates for nutraceuticals, functional foods, and biotechnological applications. Future research should focus on optimizing cultivation, scaling production, and evaluating health benefits.

## Figures and Tables

**Table 1:** Fatty acid profile identified in the total lipid extract of cyanobacteria strains *Laspinema* sp. LEGE 06078, *Rivulariaceae cyanobacterium* LEGE 06114, and *Sphaerospermopsis* sp. LEGE 00249. Values are expressed in relative abundance (%) and represent the mean of five analytical samples ± standard deviation (SD).

	Relative Abundance (%) ± SD		
Fatty Acids	*Laspinema* sp. LEGE 06078	*Rivulariaceae cyanobacterium* LEGE 06114	*Sphaerospermopsis* sp. LEGE 00249
FA 14:0	1.9 ± 0.2	2.1 ± 0.4	4.2 ± 0.5
FA 15:0 iso	1.6 ± 0.2	-	3.6 ± 0.5
FA 16:0	37.7 ± 1.8	29.0 ± 2.3	25.1 ± 3.0
FA 16:1 *n*−9	2.5 ± 0.2	2.6 ± 0.4	-
FA 16:1 *n*−7	5.9 ± 0.2	3.4 ± 0.	10.2 ± 0.8
FA 16:1 *n*−5	-	2.6 ± 0.5	-
FA 16:2 *n*−4	-	5.0 ± 0.9	-
FA 16:3 *n*−3	-	3.4 ± 0.6	-
FA 17:0	2.6 ± 0.3	-	-
FA 17:1 *n*−8	2.2 ± 0.2	-	-
FA 18:0	5.9 ± 0.7	8.5 ± 0.9	15.1 ± 1.4
FA 18:1 *n*−9	9.0 ± 0.6	10.3 ± 1.3	5.9 ± 0.4
FA 18:1 *n*−7	2.6 ± 0.2	-	-
FA 18:1 *n*−6	-	3.3 ± 0.3	6.5 ± 0.
FA 18:2 *n*−6	5.0 ± 0.4	9.5 ± 0.2	10.2 ± 1.1
FA 18:3 *n*−6	3.7 ± 0.5	12.3 ± 1.2	-
FA 18:3 *n*−3	8.9 ± 0.3	3.8 ± 0.5	11.0 ± 1.2
FA 18:4 *n*−3	10.4 ± 0.3	4.2 ± 0.4	-
FA 24:0	-	-	8.3 ± 1.0
∑ SFA	49. ± 0.6	39.6 ± 1.7	56.3 ± 0.6
∑ MUFAs	22.2 ± 0.2	22.2 ± 0.5	22.5 ± 0.4
∑ PUFAs	28.1 ± 0.7	38.2 ± 1.4	21.2 ± 0.3
∑ *n*−3	19.3 ± 0.6	11.4 ± 1.6	11.0 ± 1.2
∑ *n*−6	8.8 ± 0.9	26.8 ± 0.6	10.2 ± 10.1
*n*−6/*n*−3 ratio	0.4	2.4	0.9
AI	0.9 ± 0.03	0.6 ± 0.03	1.0 ± 0.02
TI	0.6 ± 0.02	0.7 ± 0.09	0.9 ± 0.05
(h/H)	0.6 ± 0.01	0.8 ± 0.02	0.9 ± 0.01

PUFAs: polyunsaturated fatty acids. SFA: saturated fatty acid. MUFAs: monounsaturated fatty acids. AI: atherosclerotic index. TI: thrombogenic index. (h/H): (hypocholesterolemic/Hypercholesterolemic) ratio.

**Table 2:** Polar lipid classes identified in the total lipid extracts of strains *Laspinema* sp. LEGE 06078, *Rivulariaceae cyanobacterium* LEGE 06114, and *Sphaerospermopsis* sp. LEGE 00249. The total number of species identified in each class and major lipid species per class is shown.

	Number of Lipid Species			Major Species		
Lipid Classes	LEGE 06078	LEGE 06114	LEGE 00249	LEGE 06078	LEGE 06114	LEGE 00249
Glycolipids	48	57	28			
MGDG	25	25	9	MGDG (34:4)	MGDG (34:3)	MGDG (34:4)
DGDG	12	12	10	DGDG (34:4)	DGDG (34:3)	DGDG (34:3)
DGGA	-	9	-	-	DGGA (35:1)	-
SQDG	11	11	9	SQDG (34:1)	SQDG (34:1)	SQDG (34:3)
Phospholipids	35	10	36			
PC	2	2	16	PC (36:2)	PC (36:2)	PC (36:2)
LPC	1	-	4	LPC (18:1)	-	LPC (18:1)
PG	18	6	8	PG (34:1)	PG (34:1)	PG (34:1)
LPG	-	-	1	-	-	LPG (16:0)
PE	5	2	5	PE (36:2)	PE (30:0)	PE (32:1)
LPE	-	-	1	-	-	LPE (18:1)
PI	9	-	1	PI (34:1)	-	PI (34:1)
Betaine lipids	-	4	37			
DGTS	-	4	24	-	DGTS (36:2)	DGTS (34:3)
MGTS	-	-	13	-	-	MGTS (16:0)
Neutral lipids	42	35	12			
TG	38	32	12	TG (50:1)	TG (48:0)	TG (52:2)
DG	4	3	-	DG (34:4)	DG (34:3)	-
Total	125	106	113			

MGDG: monogalactosyldiacylglycerol; DGDG: digalactosyldiacylglycerol; DGGA: diacylglyceryl-α-D-glucuronide; SQDG: sulfoquinovosyl diacylglycerol; TG: triacylglycerol; DG: diacylglycerol; LPC: lysophosphatidylcholine; LPG: lysophosphatidylglycerol; LPE: lysophosphatidylethanolamine; PC: phosphatidylcholine; PE: phosphatidylethanolamine; PG: phosphatidylglycerol; PI: phosphatidylinositol; DGTS: diacylglyceryl-N,N,N-trimethyl-homoserine; MGTS: monoacylglyceryl-N,N,N-trimethyl-homoserine.

**Table 3:** Common polar lipid species identified in cyanobacteria strains.

Glycolipids			
DGDG (32:0)	DGDG (34:4)	MGDG (34:1)	SQDG (30:0)
DGDG (32:1)	DGDG (34:5)	MGDG (34:2)	SQDG (34:1)
DGDG (34:1)	MGDG (32:0)	MGDG (34:3)	SQDG (34:3)
DGDG (34:2)	MGDG (32:1)	MGDG (34:4)	SQDG (36:0)
DGDG (34:3)	MGDG (32:2)	MGDG (36:2)	
Phospholipids			
PC (36:2)	PG (34:1)	PG (34:2)	
Neutral lipids			
TG (44:0)	TG (45:0)	TG (47:1)	TG (48:2)

## Data Availability

The original contributions presented in this study are included in the article/Supplementary Materials. Further inquiries can be directed to the corresponding author. The accession numbers of the 16S rRNA genes are as follows: *Laspinema* sp. LEGE 06078 (PP490654), *Rivulariaceae cyanobacterium* LEGE 06114 (PP477455), and *Sphaerospermopsis* sp. LEGE 00249 (PP490656).

## Supplementary Materials

The following supporting information can be downloaded at https://www.mdpi.com/article/10.3390/molecules30122504/s1: Figure S1: Agarose gel electrophoresis of toxin-producing gene PCR amplification of cyanobacterial strains. The red box indicates the cyanobacteria of this work. (M) A 1 kb Plus DNA Ladder. (+) Positive control. (-) Negative control. (a) Anatoxin; (b) Cylindrospermopsin; (c) Saxitoxin; (d) Microcystin. Figure S2: Protein content. Different letters indicate significant differences (*p* < 0.05). Figure S3: Lipid content. Different letters indicate significant differences (*p* < 0.05). Figure S4: MDS analysis of proximal composition. Figure S5: PCA of fatty acid composition. Figure S6: Complete linkage cluster analysis dendrogram showing the similarities (Euclidean distances) of FA profiles of cyanobacteria strains. Figure S7: Bar plot of top 15 of most abundant lipid species. (a) Glycolipids; (b) phospholipids; (c) betaine lipids; (d) neutral lipids. Figure S8: Development stages of *D. rerio*: (a) 0.2 h–6 h post fertilization, (b) 24 h post fertilization, (c) 120 h post fertilization. Table S1: Biomass proximate composition (%) of macronutrients of cyanobacterial strains. Table S2: Fatty acid profile identified in the total lipid extract of cyanobacteria (absolute abundance). Table S3: Lipid species identified in the total lipid extract of cyanobacteria. Table S4: Primers and PCR conditions for gene amplification of cyanotoxins. Table S5: Typical fragmentation rules used to generate the assignments. NL: neutral loss. PI: Precursor Ion.
