# Supplementary material for: Unveiling the Biotechnological Potential of Cyanobacteria from the Portuguese LEGE-CC Collection Through Lipidomics and Antioxidant and Lipid-Lowering Properties
Source: Molecules. 2025 Jun 7;30(12):2504. doi: 10.3390/molecules30122504 (PMC12196109; doi:10.3390/molecules30122504)
Supplement: Supplementary file 1 [file molecules-30-02504-s001.zip › molecules-3650691-supplementary/Supplementary Table S1.pdf]

Supplementary Table S1. Biomass proximate composition (%) of macronutrients of cyanobacteria strains

	Carbon	Hydrogen	Nitrogen	Carbohydrate	Ash	Protein	Total Lipids
Laspinema sp. LEGE 06078	43.60 ± 0.25	6.40 ± 0.05	10.07 ± 0.09	22.54	5.41 ± 0.27	62.93 ± 0.59	9.12 ± 0.86
"*Rivularia*" sp. LEGE 06114	34.51 ± 0.99	5.48 ± 0.21	4.52 ± 0.19	46.13	17.82 ± 0.95	28.22 ± 1.22	7.82 ± 0.40
Sphaerospermopsis sp. LEGE 00249	39.63 ± 0.77	6.08 ± 0.01	9.63 ± 0.09	19.49	14.07 ± 2.98	60.22 ± 0.59	6.22 ± 0.98
