# Supplementary material for: Unveiling the Biotechnological Potential of Cyanobacteria from the Portuguese LEGE-CC Collection Through Lipidomics and Antioxidant and Lipid-Lowering Properties
Source: Molecules. 2025 Jun 7;30(12):2504. doi: 10.3390/molecules30122504 (PMC12196109; doi:10.3390/molecules30122504)
Supplement: Supplementary file 1 [file molecules-30-02504-s001.zip › molecules-3650691-supplementary/Supplementary Table S2.pdf]

Supplementary Table S2. Fatty acid profile identified in the total lipid extract of cyanobacteria (*Laspinema* sp. LEGE 06078, "*Rivularia*" sp. LEGE 06114, and *Sphaerospermopsis* sp. LEGE 00249) by GC–MS. Values are expressed in absolute abundance (mg of FA. g biomass $^{-1}$) and represent the mean of five analytical samples ± standard deviation (SD).

**Fatty acids**	**Absolute abundance (mg of FA. g biomass $^{-1}$) ± SD**		
	***Laspinema* sp. LEGE 06078**	***"Rivularia"* sp. LEGE 06114**	***Sphaerospermopsis* sp. LEGE 00249**
**FA 14:0**	0.78 ± 0.05	0.56 ± 0.10	0.53 ± 0.09
**FA 15:0 iso**	0.67 ± 0.05	-	0.44 ± 0.07
**FA 16:0**	15.70 ± 1.55	7.73 ± 0.83	3.26 ± 1.26
**FA 16:1 *n*-9**	1.04 ± 0.05	0.70 ± 0.11	-
**FA 16:1 *n*-7**	2.47 ± 0.20	0.90 ± 0.10	1.31 ± 0.44
**FA 16:1 *n*-5**	-	0.68 ± 0.13	-
**FA 16:2 *n*-4**	-	1.33 ± 0.24	-
**FA 16:3 *n*-3**	-	0.89 ± 0.17	-
**FA 17:0**	1.07 ± 0.09	-	-
**FA 17:1 *n*-8**	0.93 ± 0.07	-	-
**FA 18:0**	2.44 ± 0.21	2.25 ± 0.14	1.90 ± 0.40
**FA 18:1 *n*-9**	3.76 ± 0.42	2.75 ± 0.44	0.73 ± 0.14
**FA 18:1 *n*-7**	1.06 ± 0.06	-	-
**FA 18:1 *n*-6**	-	0.88 ± 0.11	0.82 ± 0.19
**FA 18:2 *n*-6**	2.08 ± 0.11	2.52 ± 0.23	1.27 ± 0.21
**FA 18:3 *n*-3**	3.73 ± 0.35	1.02 ± 0.15	1.42 ± 0.53
**FA 18:3 *n*-6**	1.55 ± 0.15	3.29 ± 0.47	-
**FA 18:4 *n*-3**	4.32 ± 0.35	1.11 ± 0.14	-
**FA 24:0**	-	-	1.02 ± 0.17
**∑ PUFAs**	11.68 ± 0.66	10.16 ± 0.95	2.69 ± 0.69
**∑ SFA**	20.66 ± 1.26	10.54 ± 0.72	7.15 ± 1.83
**∑ MUFAs**	9.25 ± 0.53	5.91 ± 0.54	2.87 ± 0.77
**∑ *n*-3**	8.05 ± 0.68	3.02 ± 0.45	1.40 ± 0.53
**∑ *n*-6**	3.64 ± 0.25	7.15 ± 0.69	1.29 ± 0.21
***n*–6/*n*–3 ratio**	0.45	2.37	0.92
**AI**	0.90 ± 0.03	0.62 ± 0.03	0.96 ± 0.02
**TI**	0.58 ± 0.01	0.67 ± 0.09	0.77 ± 0.05
**h/H**	0.58 ± 0.01	0.76 ± 0.02	0.93 ± 0.08

PUFAs: polyunsaturated fatty acids. SFA: saturated fatty acids. MUFAs: monounsaturated fatty acids. AI: atherosclerotic index. TI: thrombogenic index. (h/H): hypocholesterolemic/hypercholesterolemic.
