# Supplementary material for: Unveiling the Biotechnological Potential of Cyanobacteria from the Portuguese LEGE-CC Collection Through Lipidomics and Antioxidant and Lipid-Lowering Properties
Source: Molecules. 2025 Jun 7;30(12):2504. doi: 10.3390/molecules30122504 (PMC12196109; doi:10.3390/molecules30122504)
Supplement: Supplementary file 1 [file molecules-30-02504-s001.zip › molecules-3650691-supplementary/Supplementary Table S4.pdf]

Supplementary Table S4. Primers and PCR conditions for gene amplification of cyanotoxins.

Amplified gene	Primer	Fragment length (bp)	Sequence (5'-3')	PCR Condition	1 cycle Initial denaturation	35 cycles Denaturation	Annealing	Extension	1 cycle Final Extension	Holding	Reference
cyrJ	cynsulF	584	ACTTCTCTCCTTTCCCTATC	Temperature	95 °C	95 °C	50 °C	72°C	72 °C	8 °C	[125]
	cylnamR		GAGTGAAAATGCGTAGAACTTG	Time	3 min	45 s	30 s	1 min	3 min	pause	
anaC	anaC-genF	366	TCTGGTATTCAGTCCCCTCTA	Temperature	94 °C	94 °C	54 °C	72 °C	72 °C	8 °C	[126]
	anaC-genR		CCCAATAGCCTGTCATCA	Time	2 min	30 s	30 s	30 s	5 min	pause	
sxtI	SxtI682F	200	AGCGCTGCCGCTATGGTTGTCG	Temperature	94 °C	94 °C	52 °C	72 °C	72 °C	8 °C	[127]
	SxtI877R		ACGCAATTGAGGGCGACACCAC	Time	3 min	10 s	20 s	1 min	7 min	pause	
Microcystin/nodularin synthetase	HEPF	472	TTTGGGGTTAACTTTTTTGGGCATAGT	Temperature	92 °C	92 °C	52 °C	72 °C	72 °C	8 °C	[128]
	HEPR		AATTCTTGAGGCTGTAAATCGGGTT	Time	2 min	20 s	30 s	1 min	5 min	pause	

REFERENCES

125. Mihali, T.K.; Kellmann, R.; Muenchhoff, J.; Barrow, K.D.; Neilan, B.A. Characterization of the Gene Cluster Responsible for Cylindrospermopsin Biosynthesis. *Appl Environ Microbiol* **2008**, *74*, 716–722, doi:10.1128/AEM.01988-07.

126. Rantala-Ylinen, A.; Känä, S.; Wang, H.; Rouhiainen, L.; Wahlsten, M.; Rizzi, E.; Berg, K.; Gugger, M.; Sivonen, K. Anatoxin-a Synthetase Gene Cluster of the Cyanobacterium Anabaena Sp. Strain 37 and Molecular Methods to Detect Potential Producers. *Appl Environ Microbiol* **2011**, *77*, 7271–7278, doi:10.1128/AEM.06022-11.

127. Lopes, V.R.; Ramos, V.; Martins, A.; Sousa, M.; Welker, M.; Antunes, A.; Vasconcelos, V.M. Phylogenetic, Chemical and Morphological Diversity of Cyanobacteria from Portuguese Temperate Estuaries. *Mar Environ Res* **2012**, *73*, 7–16, doi:10.1016/j.marenvres.2011.10.005.

128. Jungblut, A.D.; Neilan, B.A. Molecular Identification and Evolution of the Cyclic Peptide Hepatotoxins, Microcystin and Nodularin, Synthetase Genes in Three Orders of Cyanobacteria. *Arch Microbiol* **2006**, *185*, 107–114, doi:10.1007/s00203-005-0073-5.
