# Supplementary material for: Unveiling the Biotechnological Potential of Cyanobacteria from the Portuguese LEGE-CC Collection Through Lipidomics and Antioxidant and Lipid-Lowering Properties
Source: Molecules. 2025 Jun 7;30(12):2504. doi: 10.3390/molecules30122504 (PMC12196109; doi:10.3390/molecules30122504)
Supplement: Supplementary file 1 [file molecules-30-02504-s001.zip › molecules-3650691-supplementary/Supplementary Table S5.pdf]

Supplementary Table S5. Typical fragmentation rules [27] used to generate the assignments. NL: Neutral loss. PI: Product Ion.

Lipids class	Ion mode	Detected ion (MS)	Fragmentation patterns lipid class
**Glycolipids**			
Monogalactosyl diacylglycerol (MGDG)	+	$[M + NH_4]^+$	NL of 197 Da
Digalactosyl diacylglycerol (DGDG)	+	$[M + NH_4]^+$	NL of 359 Da
Sulfoquinovosyl diacylglycerol (SQDG)	-	$[M - H]^-$	PI at *m/z* 225.0
Diacylglyceryl-A-D-glucuronide (DGGA)	+	$[M + NH_4]^+$	NL of 211 Da
**Phospholipids**			
Phosphatidylcholine (PC)<br>Lyso-phosphatidylcholine (LPC)	+ and -	+ mode $[M + H]^+$<br>- mode $[M + HCOO]^-$	PI at *m/z* 184.1<br>NL of 60 Da and PI *m/z* 168
Phosphatidylglycerol (PG)<br>Lyso-phosphatidylglycerol (LPG)	-	$[M – H]^-$	PI at *m/z* 153.0, 171.0, 227.0
Phosphatidylethanolamine (PE)<br>Lyso-phosphatidylethanolamine (LPE)	-	$[M – H]^-$	PI at *m/z* 140.1
Phosphatidylinositol (PI)	-	$[M – H]^-$	PI at *m/z* 153.0, 241.0
**Betaine lipids**			
Diacyglyceryl trimethyl homoserine (DGTS)	+	$[M + H]^+$	PI at *m/z* 236.1
Monoacyglyceryl trimethyl homoserine (MGTS)	+	$[M + H]^+$	PI at *m/z* 236.1
**Neutral lipids**			
Triacylglycerol (TG)	+	$[M + NH_4]^+$	NL of FA
Diacylglycerol (DG)	+	$[M + NH_4]^+$	NL of FA

REFERENCES

27. Rey, F.; Melo, T.; Lopes, D.; Couto, D.; Marques, F.; Domingues, M.R. Applications of Lipidomics in Marine Organisms: Progress, Challenges and Future Perspectives. *Mol Omics* 2022, *18*, 357–386.
