# Supplementary figures and images for: Unveiling the Biotechnological Potential of Cyanobacteria from the Portuguese LEGE-CC Collection Through Lipidomics and Antioxidant and Lipid-Lowering Properties
Source: Molecules. 2025 Jun 7;30(12):2504. doi: 10.3390/molecules30122504 (PMC12196109; doi:10.3390/molecules30122504)

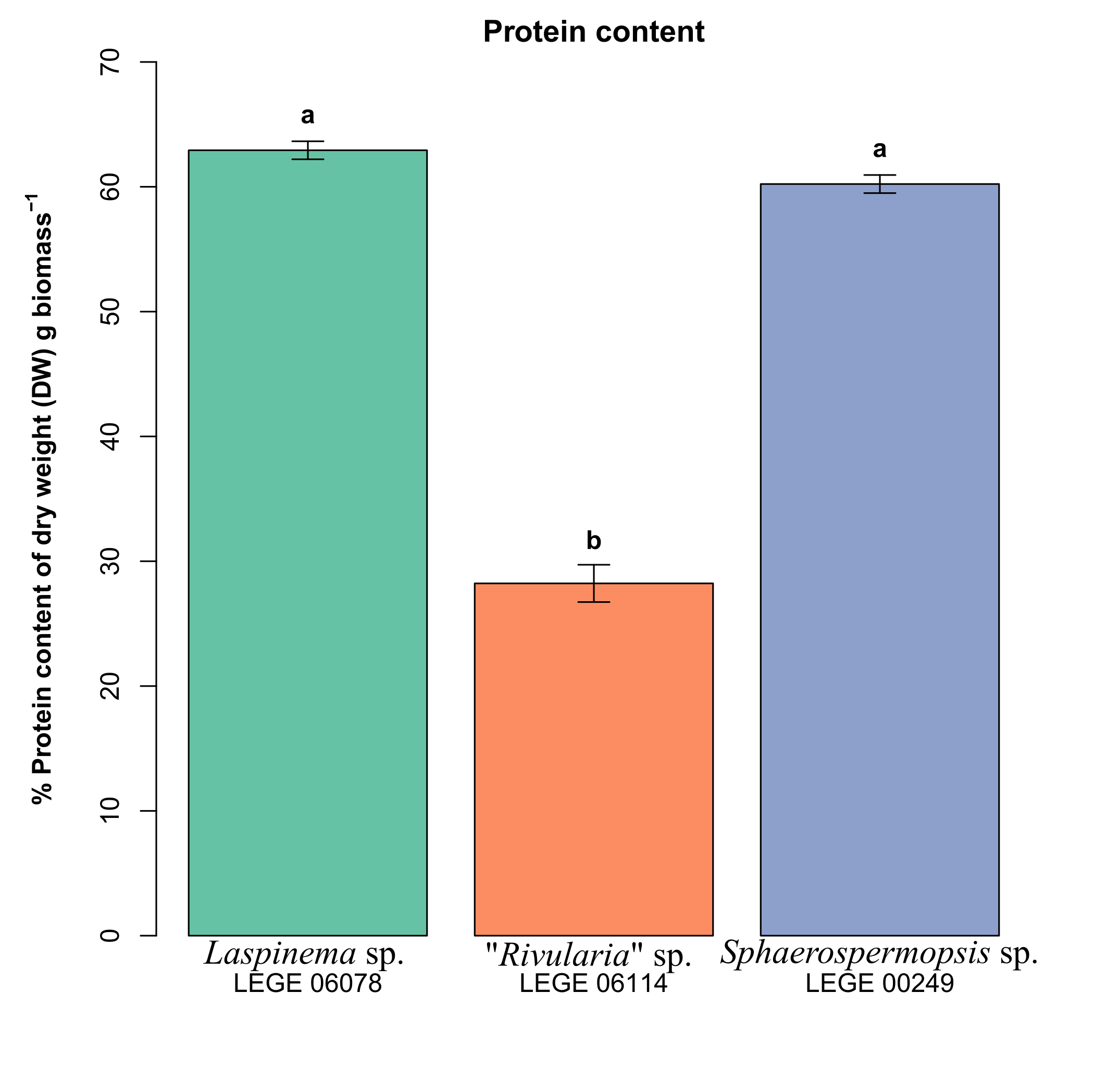

Supplement: Supplementary file 1 [file molecules-30-02504-s001.zip › molecules-3650691-supplementary/Supplementary Figure S1. Protein content.png]

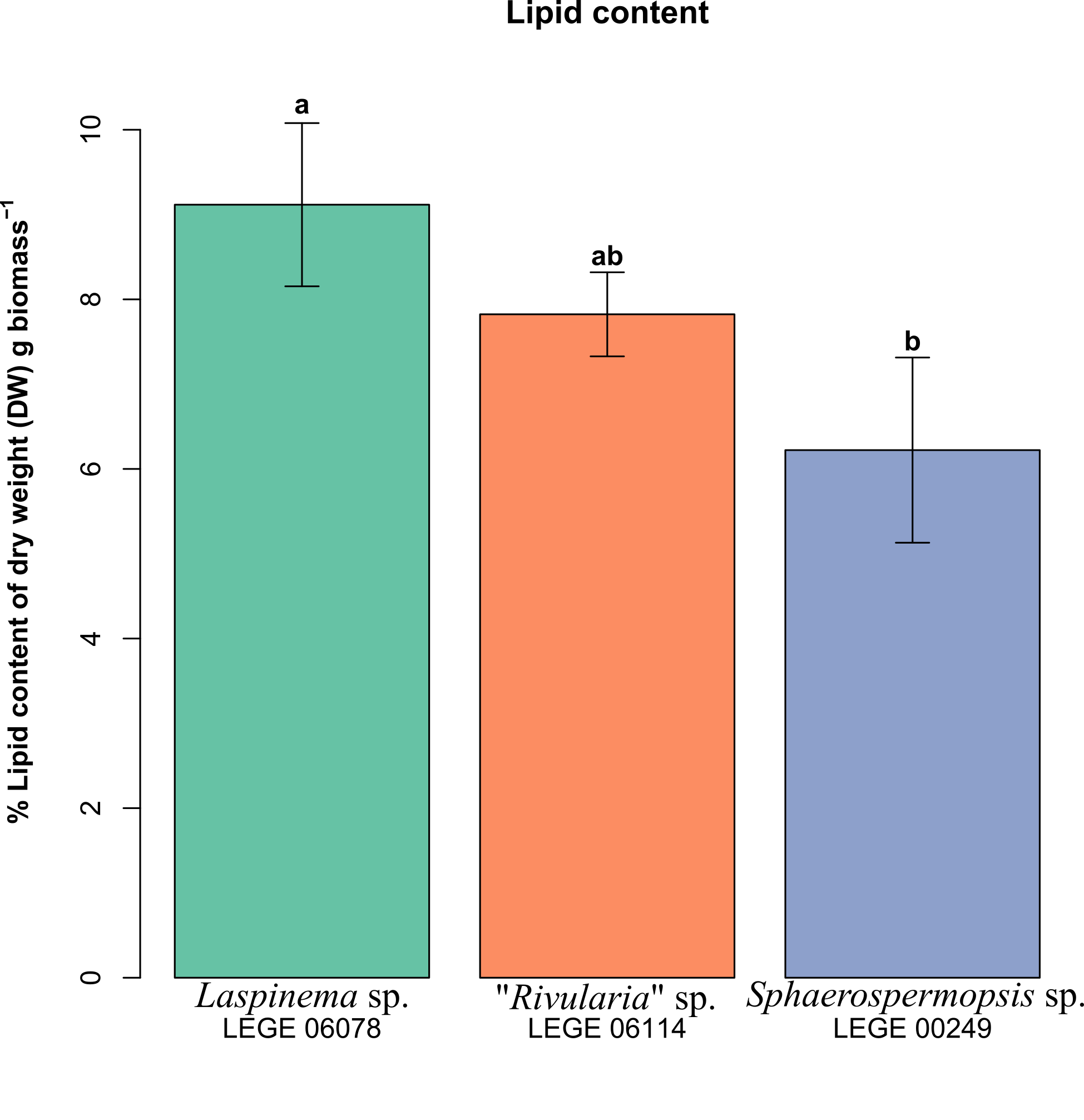

Supplement: Supplementary file 1 [file molecules-30-02504-s001.zip › molecules-3650691-supplementary/Supplementary Figure S2. Lipid content.png]

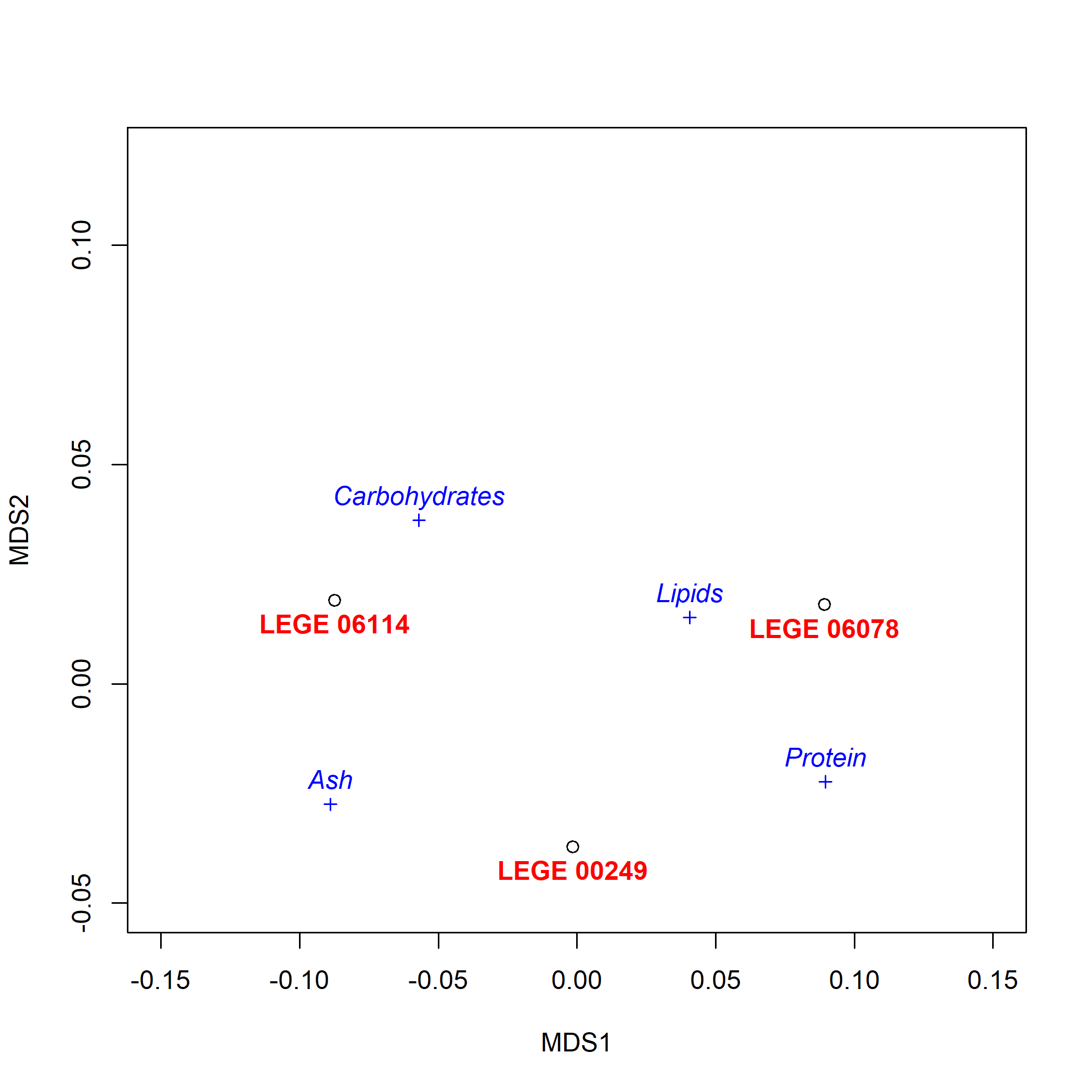

Supplement: Supplementary file 1 [file molecules-30-02504-s001.zip › molecules-3650691-supplementary/Supplementary Figure S3. MDS_Analise_elementar.tiff]

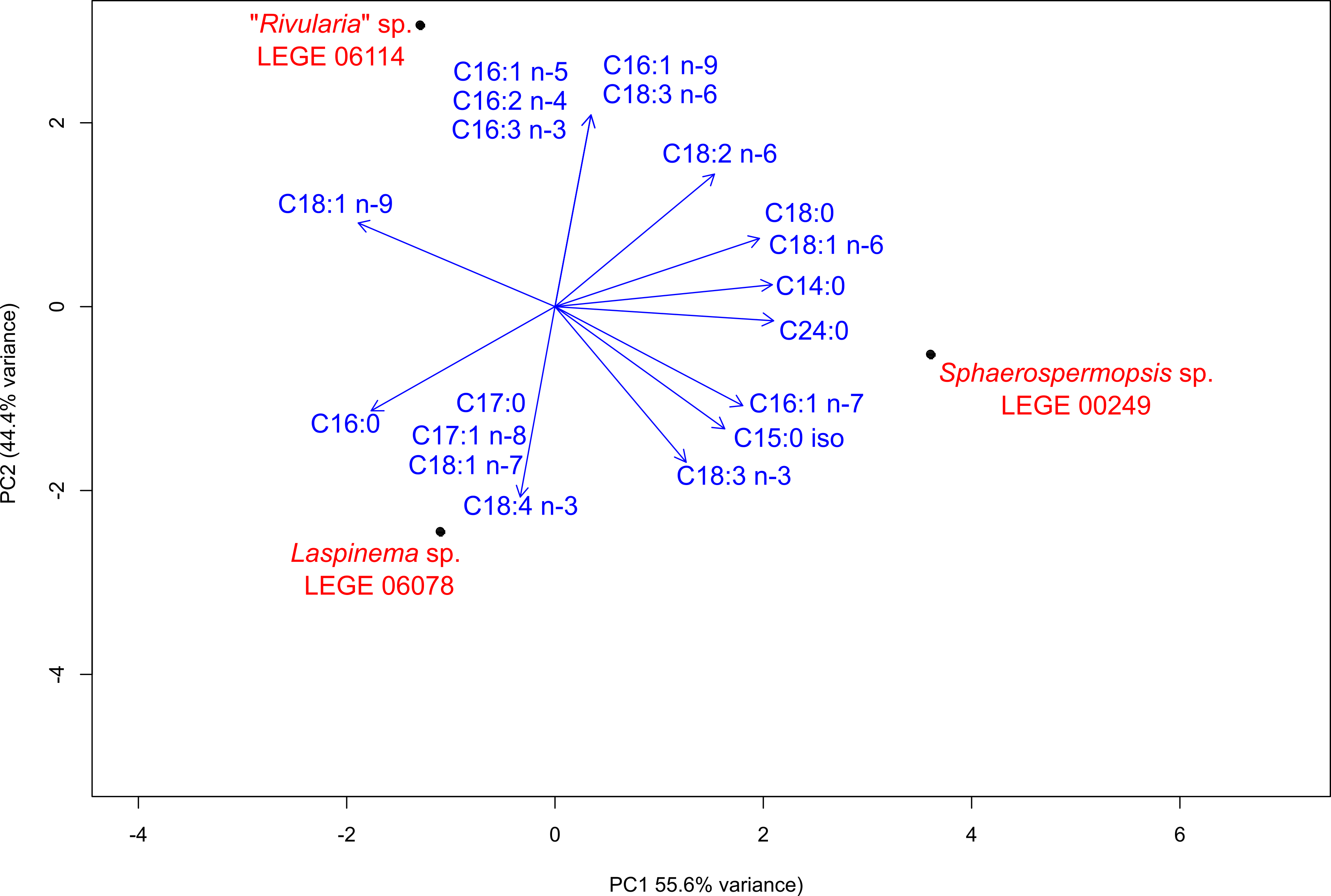

Supplement: Supplementary file 1 [file molecules-30-02504-s001.zip › molecules-3650691-supplementary/Supplementary Figure S4. PCA FAs.png]

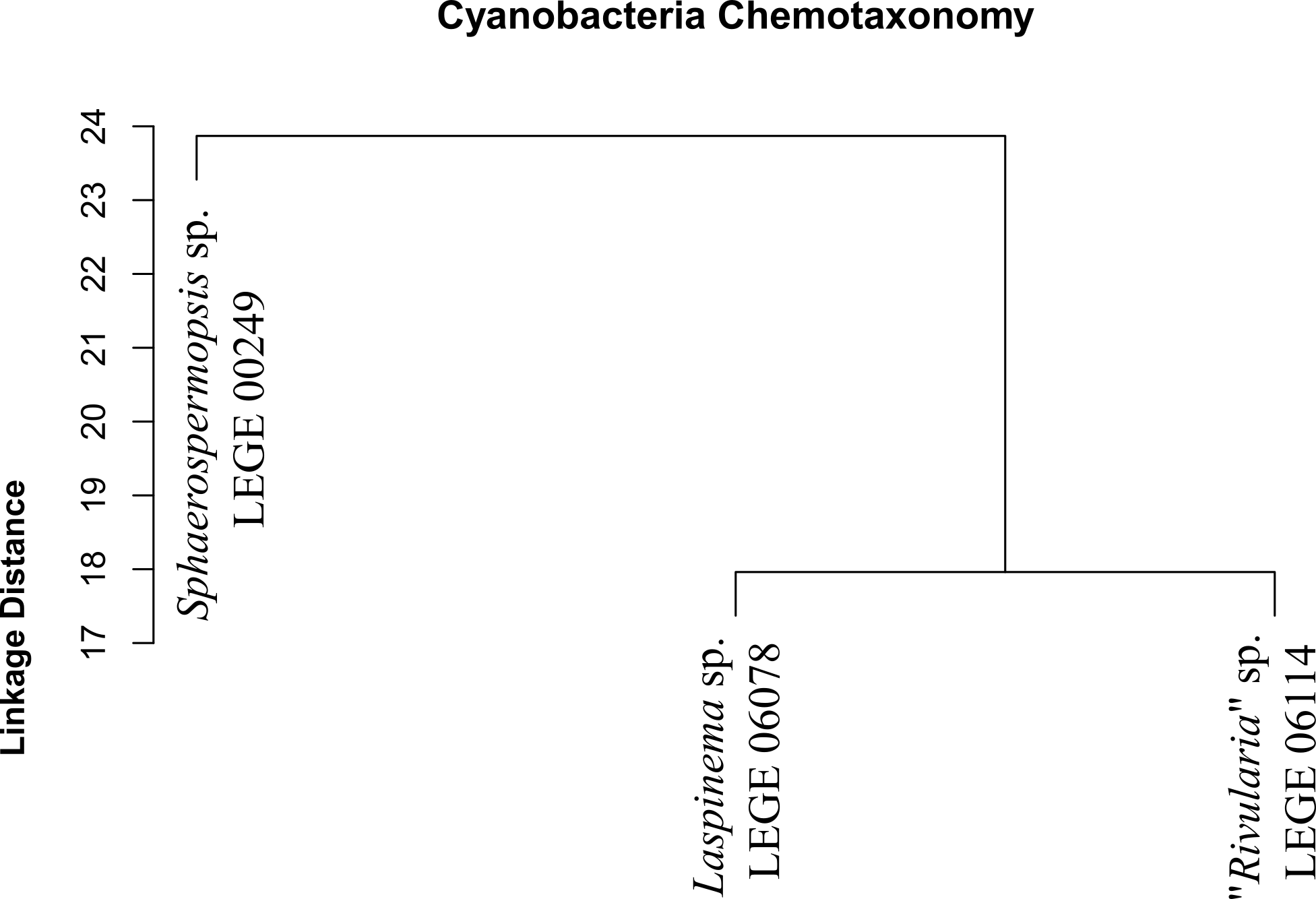

Supplement: Supplementary file 1 [file molecules-30-02504-s001.zip › molecules-3650691-supplementary/Supplementary Figure S5. Chemotaxonomy.png]

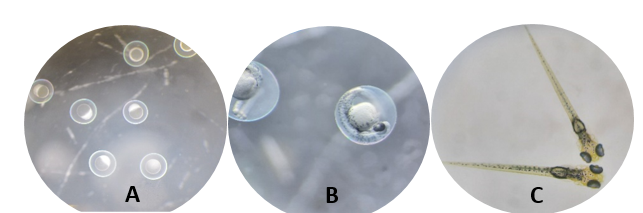

Supplement: Supplementary file 1 [file molecules-30-02504-s001.zip › molecules-3650691-supplementary/Supplementary Figure S7. Zebrafish.PNG]
